# Biodiversity on the Rocks: Macrofauna Inhabiting Authigenic Carbonate at Costa Rica Methane Seeps

**DOI:** 10.1371/journal.pone.0131080

**Published:** 2015-07-09

**Authors:** Lisa A. Levin, Guillermo F. Mendoza, Benjamin M. Grupe, Jennifer P. Gonzalez, Brittany Jellison, Greg Rouse, Andrew R. Thurber, Anders Waren

**Affiliations:** 1 Integrative Oceanography Division, Scripps Institution of Oceanography, La Jolla, California, United States of America; 2 Center for Marine Biodiversity and Conservation, Scripps Institution of Oceanography, La Jolla, California, United States of America; 3 College of Earth, Ocean, and Atmospheric Sciences, Oregon State University, 04 CEOAS Administration Building, Corvallis, Oregon, United States of America; 4 Swedish Museum of Natural History, Stockholm, Sweden; Universite Pierre et Marie Curie, FRANCE

## Abstract

***Carbonate communities*: **The activity of anaerobic methane oxidizing microbes facilitates precipitation of vast quantities of authigenic carbonate at methane seeps. Here we demonstrate the significant role of carbonate rocks in promoting diversity by providing unique habitat and food resources for macrofaunal assemblages at seeps on the Costa Rica margin (400–1850 m). The attendant fauna is surprisingly similar to that in rocky intertidal shores, with numerous grazing gastropods (limpets and snails) as dominant taxa. However, the community feeds upon seep-associated microbes. Macrofaunal density, composition, and diversity on carbonates vary as a function of seepage activity, biogenic habitat and location. The macrofaunal community of carbonates at non-seeping (inactive) sites is strongly related to the hydrography (depth, temperature, O_2_) of overlying water, whereas the fauna at sites of active seepage is not. Densities are highest on active rocks from tubeworm bushes and mussel beds, particularly at the Mound 12 location (1000 m). Species diversity is higher on rocks exposed to active seepage, with multiple species of gastropods and polychaetes dominant, while crustaceans, cnidarians, and ophiuroids were better represented on rocks at inactive sites. Macro-infauna (larger than 0.3 mm) from tube cores taken in nearby seep sediments at comparable depths exhibited densities similar to those on carbonate rocks, but had lower diversity and different taxonomic composition. Seep sediments had higher densities of ampharetid, dorvilleid, hesionid, cirratulid and lacydoniid polychaetes, whereas carbonates had more gastropods, as well as syllid, chrysopetalid and polynoid polychaetes. ***Stable isotope signatures and metrics*: ** The stable isotope signatures of carbonates were heterogeneous, as were the food sources and nutrition used by the animals. Carbonate δ^13^C_inorg_ values (mean = -26.98‰) ranged from -53.3‰ to +10.0‰, and were significantly heavier than carbonate δ^13^C_org_ (mean = -33.83‰), which ranged from -74.4‰ to -20.6‰. Invertebrates on carbonates had average δ^13^C (per rock) = -31.0‰ (range -18.5‰ to -46.5‰) and δ^15^N = 5.7‰ (range -4.5‰ to +13.4‰). Average δ^13^C values did not differ between active and inactive sites; carbonate fauna from both settings depend on chemosynthesis-based nutrition. Community metrics reflecting trophic diversity (SEAc, total Hull Area, ranges of δ^13^C and δ^15^N) and species packing (mean distance to centroid, nearest neighbor distance) also did not vary as a function of seepage activity or site. However, distinct isotopic signatures were observed among related, co-occurring species of gastropods and polychaetes, reflecting intense microbial resource partitioning. Overall, the substrate and nutritional heterogeneity introduced by authigenic seep carbonates act to promote diverse, uniquely adapted assemblages, even after seepage ceases. The macrofauna in these ecosystems remain largely overlooked in most surveys, but are major contributors to biodiversity of chemosynthetic ecosystems and the deep sea in general.

## Introduction

While much of the deep sea is covered with mud, hard-substrate communities develop on steep canyon walls, seamounts, mid-ocean ridges, coral mounds, manganese crusts and nodule fields. They also abound at chemosynthetic ecosystems, where hard-substrate assemblages form on authigenic carbonates at methane seeps [1], on sulfide precipitates at hydrothermal vents [2], and on whale bone or wood at organic falls [3]. Carbonate precipitation at seeps is a by-product of anaerobic oxidation of methane [4] carried out by microbial consortia of sulfate reducing bacteria and methane oxidizing archaea [5] according to the equation [6]:

$${\text{CH}}_{4}+{\text{SO}}_{4}{}^{2}\to {\text{HCO}}_{3^{-}}+{\text{HS}}^{-}+{\text{H}}_{2}\text{O}$$ (1)

Alkalinity is produced and the increase in subsurface alkalinity combined with high pH leads to carbonate supersaturation and precipitation.

$${\text{Ca}}^{2+}+2{\text{HCO}}_{3^{-}}\to {\text{CaCO}}_{3}+{\text{CO}}_{2}+{\text{H}}_{2}\text{O}$$ (2)

Bicarbonate produced by anaerobic methane oxidation (AOM) combines with dissolved cations to precipitate various forms of carbonate. This process can create rocks, pavements, slabs, and chemoherms—massive microbial reefs [7; 8; 9; 10; 11]. Authigenic carbonates may represent an important global carbon reservoir in the past [12] as well as the present, sequestering approximately 14% of methane in active seep settings, as well as CO_2_ which would otherwise be emitted from the seafloor [13; 14; 15]. Carbonates are broadly distributed on continental margins and in fossil seeps on land e.g., [16; 17], and typically have carbon isotopic values that are much more negative (lighter) than surrounding bottom-water values due to the incorporation of δ^13^C-depleted methane-derived carbon [9; 10; 18; 19; 20]. They are present at nearly all of the known methane seep sites in the Pacific [7; 10], as well as globally [11] and citations within. Massive AOM-based carbonate formations are well described in the eastern Pacific Ocean from the margins of Alaska [18], Oregon [7], California [8], Costa Rica [10] and Chile [21]. More recently they have been reported from serpentinite-hosted [22] and off-axis hydrothermal vents [23].

The associations of macrofauna with carbonate have been studied for coral reefs, but are less known in deep-sea ecosystems. Common forms of association include loosely associated mobile megafauna, surface-attached epifauna, endofauna (those that live in burrows and crevices within the rock) and endolithofauna (organisms dwelling within the mineral lattice). Seep tubeworms (Siboglinidae) appear to facilitate precipitation and can live partially embedded in the rocks [24]. For smaller organisms, authigenic carbonates may provide substrate for settlement and attachment, reproductive sites, refuge from predators and, in some cases, a food supply. The community structure and ecological roles of carbonate macrofauna within seep ecosystems and their links to AOM have yet to be addressed. We set out to study these ecosystems at methane seeps with the hypothesis that diverse epi- and endofauna may rely on AOM for substrate settlement, refuge and nutrition.

Previous quantitative studies of seep ecosystems have focused mainly on large structure-forming megafaunal invertebrates and their associated invertebrate communities e.g., [25, 26; 27; 28; 29] or on soft-sediment assemblages reviewed in [30; 31]. Jensen et al. [32] provided an ecological description of seep carbonate biota in shallow ‘bubbling reefs’ off the Danish coast. They documented over 100 species of macrobenthos and aggregations of crabs and lobsters associated with carbonate-cemented slabs and pillars. Stable isotope analyses did not reveal unusually light δ^13^C signatures characteristic of methane influence; most animal tissues had δ^13^C values of –17 to –24‰. This result, common for shallow water seeps e.g., [33; 34; 35], contrasts with findings for sediment fauna at deep seeps e.g., [36; 37]. Hard-bottom communities can be influenced by reduced fluids that bathe the substrate from beneath. The East Flower Garden (Gulf of Mexico) faunas on deep carbonate reefs are bathed by sulfidic, saline fluids overflowing from a brine lake. They exhibit enhanced densities and predominance of sipunculans, oligochaetes and eunicid, nereidid, spionid and sabellid polychaetes [38]. Note these observations were made before the first account of chemosynthetic seep communities [39], and thus were not interpreted in the context of seep ecosystems.

Descriptions of seep carbonate biota are slowly emerging. Ritt et al. [1] analyzed three pieces of carbonate from 1111 m at seeps in the Marmara Sea and found elevated biomass and density relative to sediment faunas, attributable largely to gastropods and mussels [1]. Examination of three rocks from each of three locations in the Nile Delta suggested low faunal densities (relative to reduced sediments) and high spatial heterogeneity, even between rocks [40]. Macrofauna on six rocks from the Del Mar Seep off southern California exhibited lower densities but higher diversity than in adjacent seep sediments [41]. Gaudron et al. [42] deployed carbonates along with organic substrates near three different reducing habitats including cold seeps in the eastern Mediterranean, a mud volcano in the Norwegian Sea, and hydrothermal vents on the Mid-Atlantic Ridge for durations of 2 weeks to 1 year. Colonization of the four carbonate rocks (one in each setting) was limited, with densities much lower than on the wood and alfalfa substrates deployed. Similar deployment of carbonate cubes at 350–1100 m for 1–2 years on three mud volcanoes in the Gulf of Cádiz also yielded low densities of macrofauna (35 ind. dm^-3^) relative to organic substrates, and high evenness [43]. All of these studies hint at tremendous variability in seep carbonate biota related to proximity to seepage.

### Objectives

This study was conducted to assess how carbonate ecosystems contribute to both species diversity and trophic diversity on continental margins and to identify the factors that drive this diversity. To date, seep carbonate macrofauna (animals retained on a 0.3 mm mesh) are poorly known or undescribed at most sites. Here we characterize the abundance, composition, diversity and trophic attributes of invertebrate faunas associated with authigenic carbonates at methane seeps on the Costa Rica margin. We assess whether these carbonate community attributes are (1) similar on carbonates at sites experiencing active seepage relative to those experiencing apparent inactivity, (2) affected by the hydrography of overlying water (temperature, oxygen, water depth) and by different locations on the Costa Rica margin, and (3) differ in the presence of varied biogenic habitats (bathymodiolin mussel beds, siboglinid tubeworm bushes, microbial mats and clam beds). We hypothesized (*a priori*) that the microbes responsible for precipitating the carbonates via AOM may provide a food source for the carbonate fauna and that fauna at the more active sites should exhibit higher densities, lower diversity associated with greater food supply, and greater reliance on AOM as reflected in lighter δ^13^C signatures. The majority of macrofaunal studies at methane seeps have addressed sediment communities (infauna) [30; 31]. We compared the density, composition and diversity of Costa Rica carbonate communities to those of infaunal seep assemblages sampled in close proximity to better understand the role of substrate in generating biotic heterogeneity. By identifying the factors that control the distribution of fauna associated with authigenic carbonates, we contribute significantly to knowledge of this ubiquitous but long overlooked habitat, and to our understanding of the biodiversity of our continental margins.

### Costa Rica seeps and their carbonates

At the Costa Rica margin the Cocos Plate subducts beneath the Caribbean Plate at a rate of 90 mm per year [44]. As a result, expulsion of methane-rich fluids and gases occurs over extended areas ranging in depth from 730–3800 m [45; 46]. Active seepage occurs in diverse margin settings including at landslides, scarps associated with seamount subduction, fault intersections and mid-slope mud volcanoes [46; 47]. Carbonate precipitation usually occurs at the sulfate-methane transition zone, close to the sediment/water interface, at temperatures similar to that of bottom water [10]. The Costa Rica carbonates at sites between 800 and 1500 m have been reported to be depleted in δ^13^C (as light as -53‰), reflecting both thermogenic and biogenic methane sources [10]. Microscopic, geochemical and isotope analysis of 300 pieces of authigenic carbonates from Costa Rica mounds and slumps led Han et al. [10] to describe five types of authigenic carbonates formed by AOM with most having various proportions of HMC (high magnesium calcite) and aragonite.

**Fig 1:**
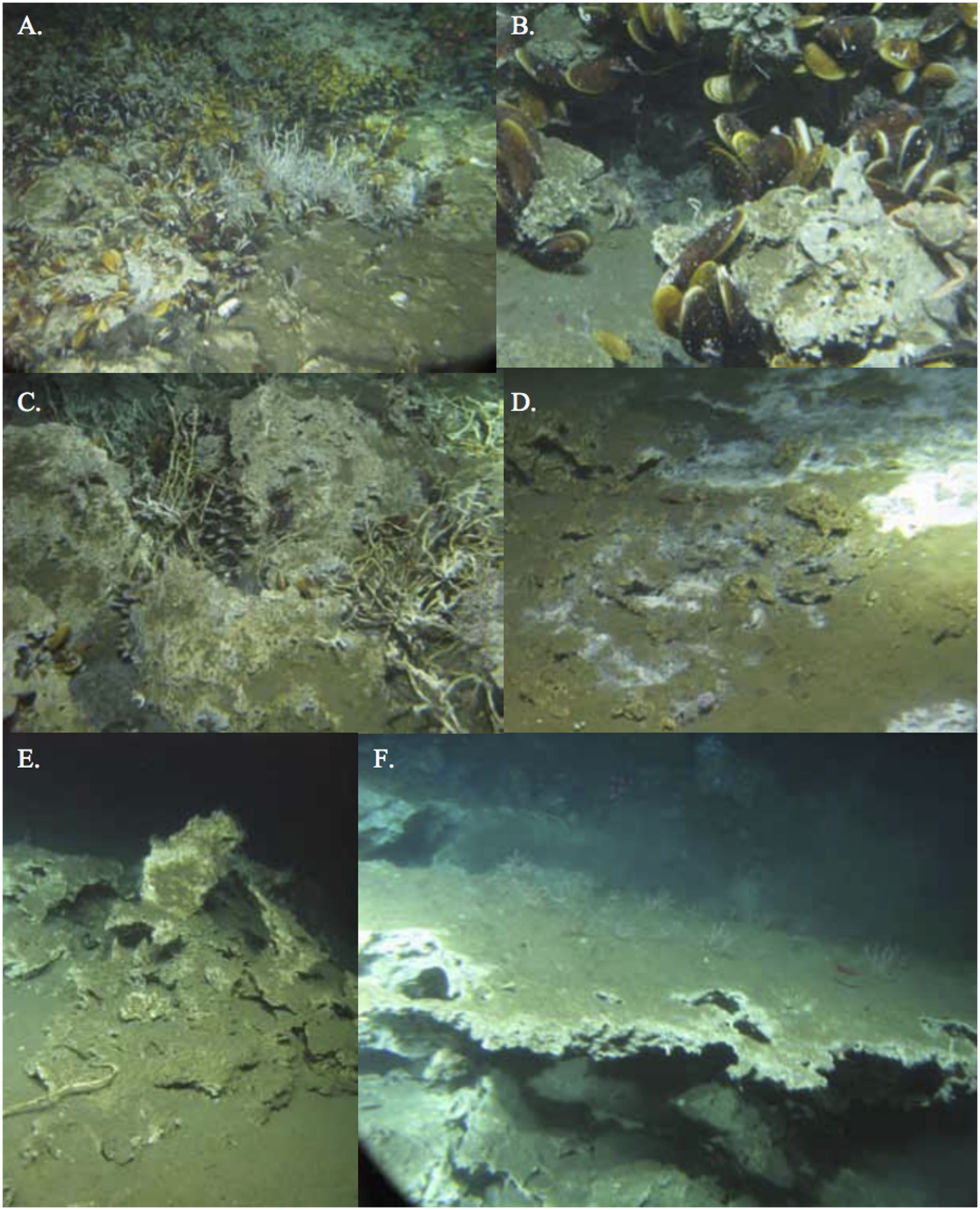
Carbonates formations in different habitats on the Costa Rica Margin. Active seepage: A. Mussel bed and tubeworm habitat; B. Mussel Bed; C. Tubeworm habitat; D. Bacterial mat; Inactive Sites E, F.

Seep sites on the Costa Rica margin have been studied with respect to bathymetry and landscape structure including large chemosynthetic biota [46; 48], carbonate, gas hydrate and sediment structure and geochemistry [10; 49], fluid flux [47; 50; 51] and methane flux [52]. Sahling et al. [48] report clusters of siboglinid tubeworms, aggregations of vesicomyid clams, beds of bathymodiolin mussels, and bacterial mats at multiple seep sites associated with subducting seamounts. Many of these are associated with carbonate rocks, boulders and mounds (Fig 1). At 1850 m on Jaco Wall, a subducting seamount on the Costa Rica margin, Levin et al. [53] document a hydrothermal seep where methane-rich fluids warmer than ambient temperature support assemblages of species associated with hydrothermal vent *and* cold seep ecosystems.

**Fig 2:**
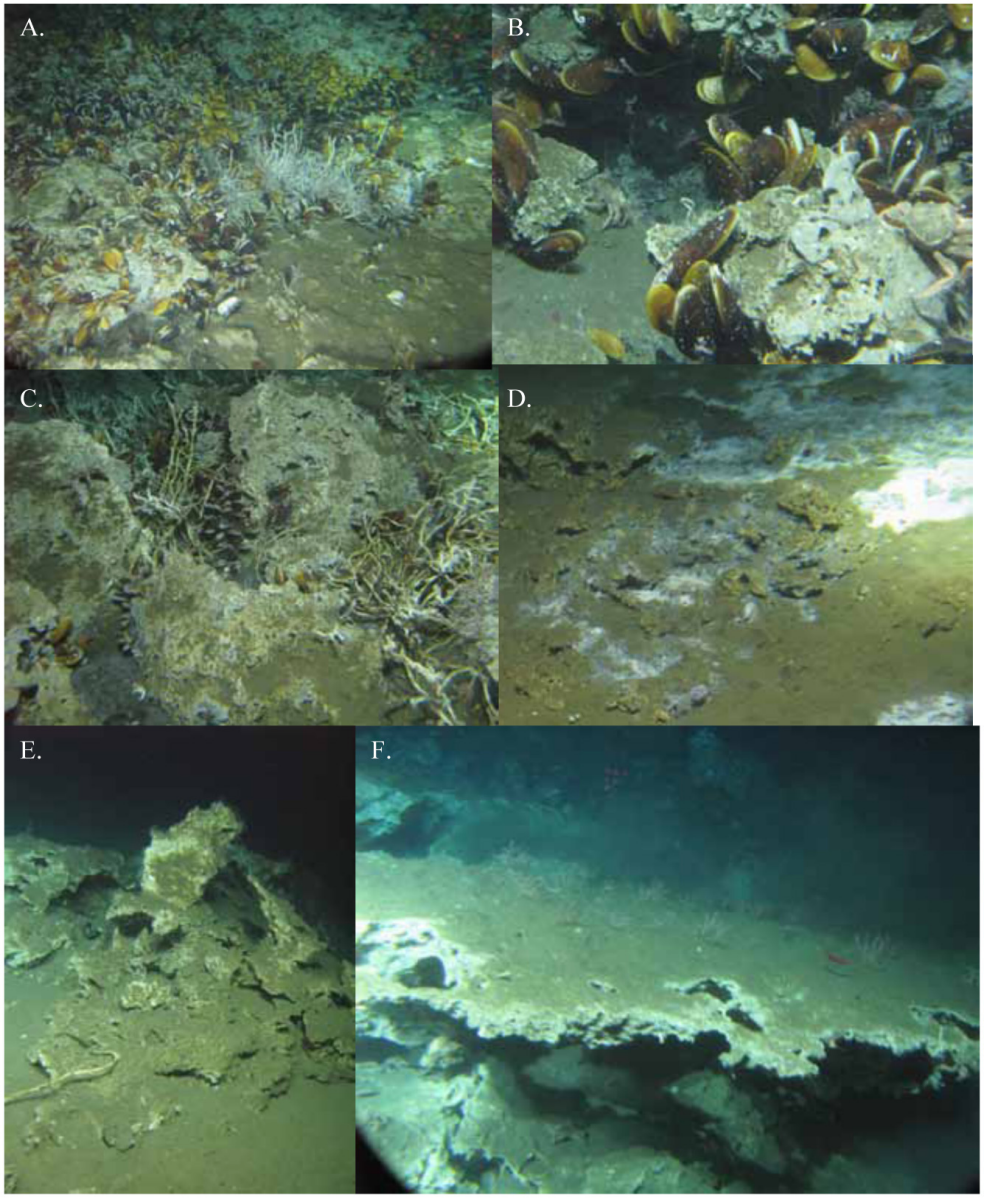
Temperature and dissolved oxygen profile generated near Jaco Wall, Costa Rica from a CTD cast.

The Costa Rica margin hosts strong vertical hydrographic gradients. Between 400 m and 1800 m the temperature ranges from 9.5 to 2.7°C, bottom-water O_2_ concentration varies from 0.04 to 1.6 ml l^-1^, and pH ranges from 7.7 to 7.8. A well-developed oxygen minimum zone (OMZ) intercepts the Costa Rica margin between 300 and 700 m (Fig 2).

## Methods

### Sampling

**Fig 3:**
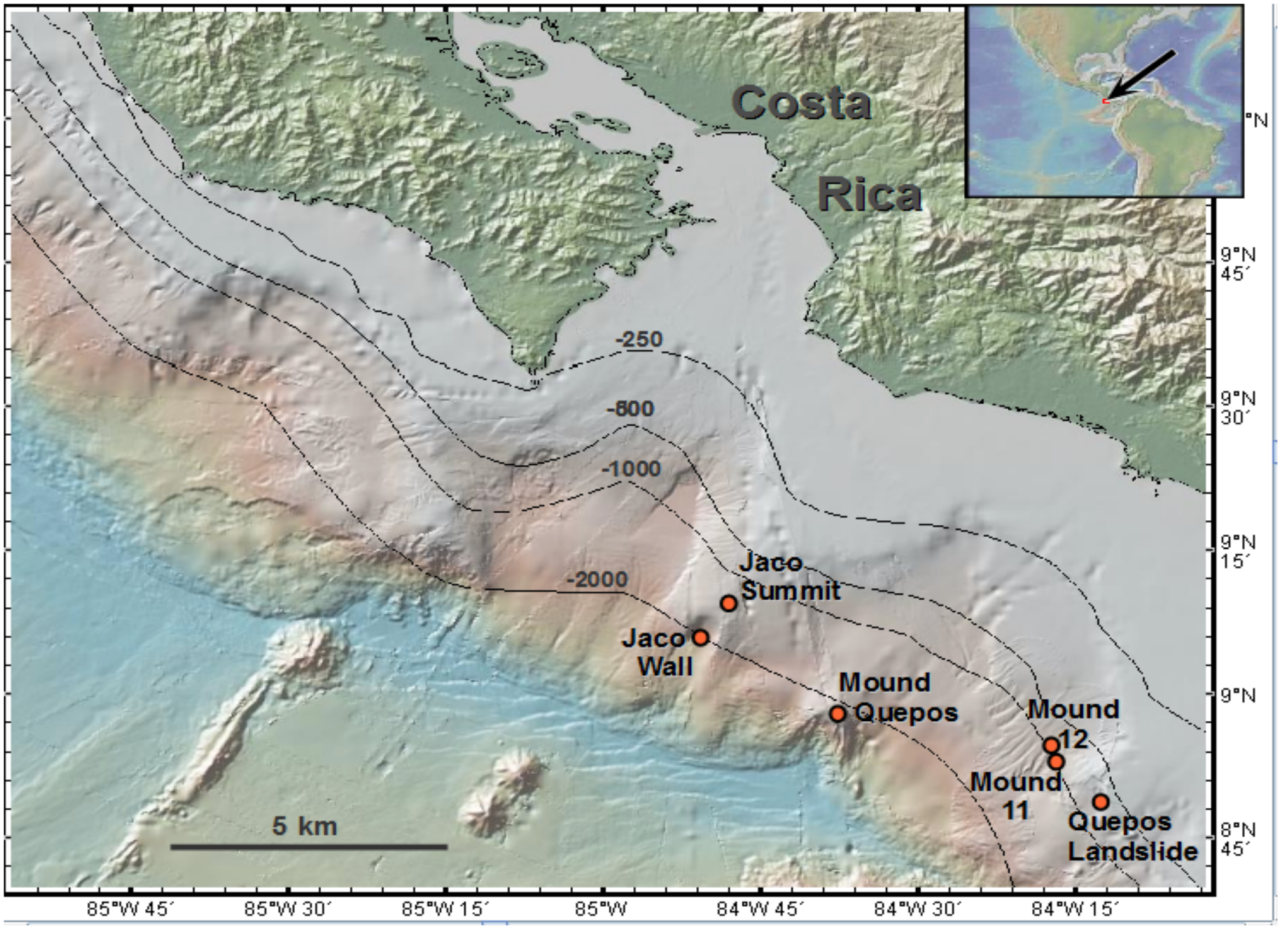
General location of seep carbonate and sediment sampling on the Costa Rica Margin.

All samples were obtained in accordance with a collecting permit issued by the Costa Rica Ministerio del Ambiente y Energía, Sistema Nacional de Áreas de Conservación. No vertebrate samples were taken. Sampling of authigenic carbonates and seep sediments took place aboard the RV Atlantis (AT 15–44) using the submersible *Alvin* from 22 February to 7 March 2009 on the Costa Rica margin. Thirty-eight rocks were collected with a robotic manipulator and placed into individual containers of a multi-compartment biobox formed from thick delrin. Rocks were collected from active and inactive locations within each site when possible (Table 1). Activity level was defined visually by presence of microbial mat development, methane bubbles or seep megafauna (bathymodiolin mussels, vesicomyid clams, and/or siboglinid tube worms) (Fig 1A–1D). Inactive sites typically involved the absence of any of the above features (Fig 1E and 1F). Samples were taken from six locations: Quepos Landslide, Mound 11, Mound 12, Mound Quepos, Jaco Wall and Jaco Summit (Fig 3; Table 1). The stations are located within the OMZ where there is < 0.5 ml l^-1^ O_2_ (Quepos Landslide; 400 m), at the lower boundary of the OMZ (Jaco Summit; 740m), and below the OMZ (Mounds 11, 12, Quepos, and Jaco Wall; 990–1854 m; Fig 2). Sediment macrofauna were sampled by tube cores (6.4 cm inside diameter) using the *Alvin* manipulator. A total of 20 cores were collected from microbial-mat covered or vesicomyid clam bed sediments on Quepos Landslide, Mound 11, Mound 12, and Jaco Wall (Table 1).

**Table 1:** Summary of site characteristics and carbonate rock collections.

Site	Dates (2009)	ALVIN DiVES	Latitude	Longitude	Water Depth (m)	Temperature (°C)	Oxygen (ml/l) [Winkler]	pH	No. Rocks (active/inactive)	No. Tubecores
Quepos Landslide	Mar. 5	4512	9° 1.2'N	84° 30.0'W	376–411	9.5	0.26	7.7	0/5	6
Jaco Summit	Mar. 3	4510	9° 10.36'N	84° 47.93'W	741–742	6	0.38	N/A	0/2	0
Mound 12	Feb. 22–24, Mar.5	4501, 4502, 4503, 4511	8° 55.8'N	84° 18.7'W	990–997	5.11	0.99–1.60	7.6–7.7	14/3	7
Mound 11	Feb. 25, 26	4504, 4505	8° 55.3'N	84° 18.21'W	1007–1025	4.19	1.12–1.28	7.6–7.7	4/5	3
Mound Quepos	Feb. 27, Mar. 1	4506, 4508	9° 1.92'N	84° 37.22'W	1030–1402	4.14	1.4–1.5	7.8	1/1	0
Jaco Wall	Mar. 2	4509	9° 7.23'N	84° 50.53'W	1459–1854	2.69	1.89	7.76	1/2	4

CTD casts were made above each *Alvin* sampling site and T, salinity, pressure and O_2_ were recorded. Rosette water samples taken 5 meters above the bottom were subjected to Winkler titrations [54] to determine bottom-water oxygen concentrations in the vicinity of rock and tube core samples taken at each location.

### Shipboard and laboratory processing

**Fig 4:**
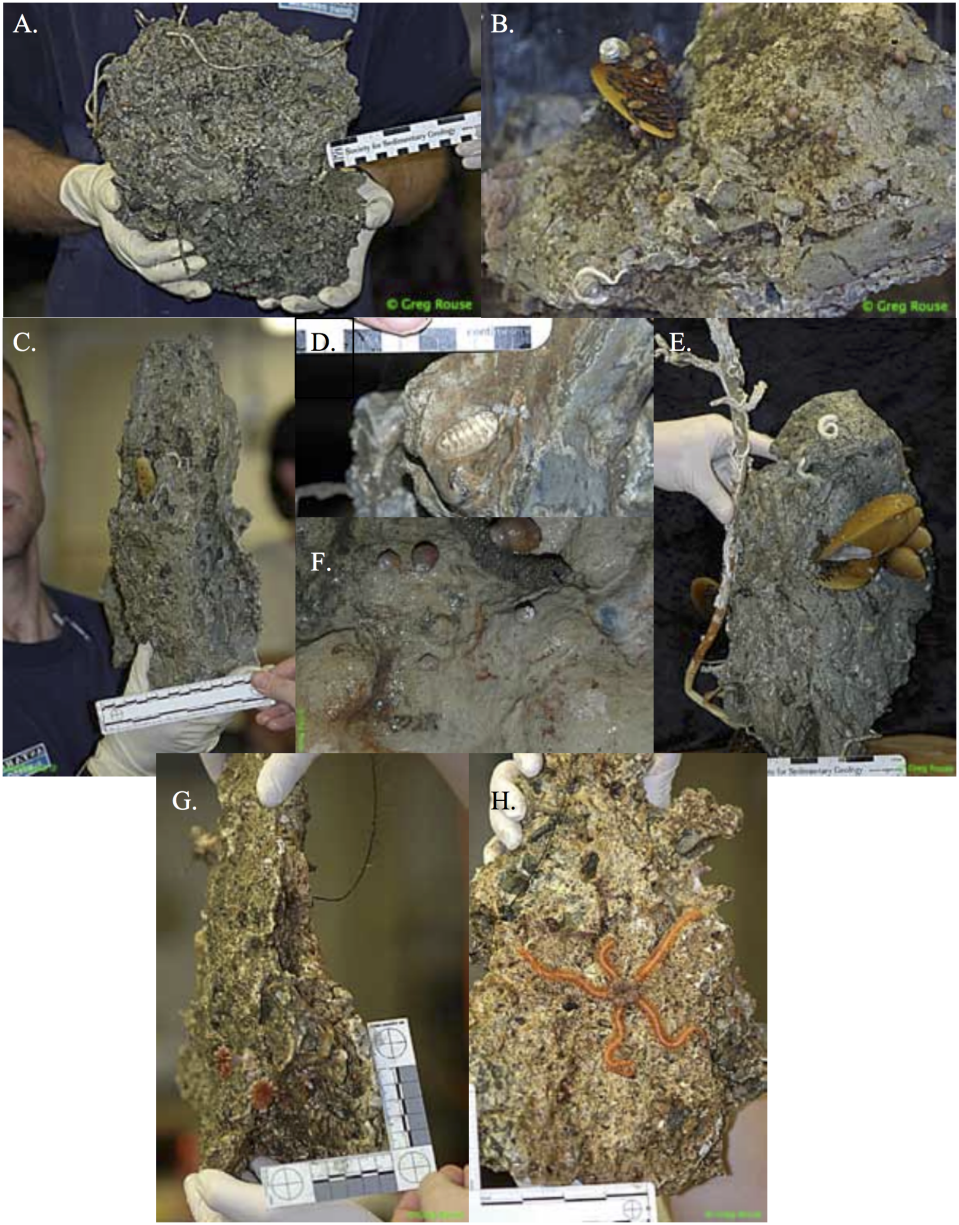
Representative carbonates and their biota from the Costa Rica Margin. Rocks were photographed soon after recover on board ship. A-F from actively seeping sites. G and H are from an inactive site.

On the ship, carbonates were photographed intact (Fig 4) and visible fauna were removed. Rock surfaces were then washed and the material sieved (to retain metazoan macrofaunal organisms > 0.3 mm). Carbonates were left in water-filled tubs at room temperature overnight to allow remaining fauna to crawl out, and were washed again on a 0.3 mm sieve the next day. Metazoans were sorted using a dissecting microscope and either (a) identified and frozen at -80°C for stable isotope analyses, or (b) preserved in 8% buffered formalin. The surface area was determined for carbonate rocks by wrapping them in a monolayer of aluminum foil. The total weight of the foil was then divided by the average weight of a 1 cm^2^ piece of foil to determine the surface area. Carbonates were air-dried and subsampled for organic and inorganic δ^13^C analyses. Tube core sediments were sectioned on board ship at 0–1, 1–2, 2–3, 3–5, and 5–10 cm intervals. The resulting macro-infaunal samples were preserved unsieved in 8% buffered formalin.

In the laboratory at Scripps Institution of Oceanography, faunal carbonate and tube core samples were washed on a 0.3 mm mesh and sorted in freshwater at 12x magnification, with identification to the lowest taxonomic level possible.

### Statistical Analyses

Macrofaunal density as well as diversity indices [Shannon diversity index (H’ log_10_), Pielou’s evenness (J’), rank 1 dominance and species richness (S)] were used to describe community structure on carbonates at each site. Faunal densities on carbonates and within sediment tube cores were standardized to number of individuals per 200 cm^-2^ surface area for comparisons, as this represented a typical rock size. Species richness was examined using species counts and rarefaction curves to compare samples with different number of individuals. Density and richness data were tested for normality and were log- or square-root transformed to achieve normality prior to analysis.

Multivariate analysis was used to identify the relationships of seepage activity (active vs inactive), habitat (bathymodiolin mussel bed, siboglinid tubeworms, vesicomyid clam bed, microbial mat, inactive), site (Quepos Landslide, Jaco Summit, Mound 12, Mound 11, Quepos Mound, Jaco Wall), and water depth (5 categories: 340–400 m, 740 m, 995–1050 m, 1400 m, 1710–1850 m) on infaunal community structure. Assemblages from actively seeping sediment habitats (microbial mats, clam beds) were compared to carbonate assemblages at overlapping sites (Quepos Landslide, Mound 12, Mound 11, Jaco Wall) as a group (all data), and as a function of site or habitat. All multivariate and ordination analyses were performed using PRIMER v.6 [55] and the add-on PERMANOVA+ module [56]. Prior to analysis, abundance data were fourth-root transformed and Bray-Curtis similarity matrices were used for resemblance-based methods. Community composition was explored following the methodology in Clarke et al. [57]. Multidimensional analysis (MDS) was used to visualize the similarity of rock fauna by activity, habitat and site.

We used a 2-way ANOVA to test whether location (Mound 11 and 12) and activity influenced macrofaunal diversity on carbonates. None of the other locations had sufficient replication of rocks at active and inactive sites for this analysis. To assess influences on community structure we used a 2-way PERMANOVA based on a Bray-Curtis similarity matrix with location and activity as independent factors. A one-way ANOVA and PERMANOVA were also performed to draw comparisons between active and inactive locations (omitting those two sites where we did not have both), recognizing that this analysis omits variability driven by site and thus is more exploratory in nature. Taxa that distinguished assemblages by site and location were identified using a percent similarity procedure (SIMPER) analysis.

A RELATE test [55] was used to test for correlations between environmental variables and macrofaunal composition. To tease apart the relative role of underlying vs overlying (hydrographic) driving factors we examined the contributions of the isotopic composition of the rock carbon (both δ^13^C_inorg_ and δ^13^C_org_) and the overlying oceanographic regime (O_2_, temperature, depth) in explaining variance in community structure. For this we used a distance-based ordination analysis [58; 59] to assess the relationship between community data and environmental and stable isotopic variables [δ^18^O (‰),δ^13^C_inorg_ (‰), δ^13^C_org_ (‰), bottom-water oxygen concentration, water depth, temperature, habitat]. The constrained ordination method of distance-based redundancy analysis (dbRDA) with the distance-based linear model (DISTLM) using the BEST AIC procedure were used to identify those variables that best explain community variation. We ran this both on the 'full model', which included all data, as well as on a reduced model, which just included samples where both active and inactive sites were present.

We used ANOVA and PERMANOVA analysis as treated above to examine contributions of biotic habitat (as characterized by the visible megafaunal assemblages) to macrofaunal community structure on carbonates. Megafauna are often treated as defining characteristics of soft sediment and on hard substrate habitats in other environments. To define whether macrofaunal assemblages in carbonate habitats are distinct from those in soft sediment seep habitats, we compared the carbonate fauna to those found in adjacent soft sediment habitats, including clam beds, microbial mats, and background communities.

A series of analyses of similarity (ANOSIM) were used to statistically account for differences between the treatments. SIMPER was applied to determine which species contributed to the observed compositional patterns. A permutational multivariate ANOVA (PERMANOVA) was used to perform a 2-factor mixed model design based on the activity level [59].

### Stable isotope methods

#### Fauna

Representative specimens of each species were removed from carbonate rocks immediately after recovery and identified to the lowest taxonomic level possible. Specimens were allowed to clear gut contents overnight in filtered seawater, washed in milli-Q water, placed in pre-weighed tin capsules or sterilized glass vials (combusted at 500°C for 4 hours) and frozen at -20 or -80°C. When specimens were large enough the remaining portion of the organism was preserved in formalin for finer taxonomic identification. In the laboratory, specimens were oven-dried (60°C), weighed and acidified with 1% PtCl_2_ in 1N HCl to remove inorganic C. Stable isotope measurements (δ^13^C, δ^15^N) were made on 0.2–1 mg of dry weight, usually from single individuals.

#### Carbonate

Rock chips were taken from each unit and oven-dried, then powdered. For measurement of δ^13^C_inorg_ and δ^18^O the powder was treated with 100% phosphoric acid for 24 hrs at 25°C (at the Scripps Institution of Oceanography) or heated to 90°C inside a vacutainer tube flushed with helium then treated with 100% phosphoric acid (at Washington State University) and the resulting released CO_2_ was analyzed on either the Thermo Finnigan Delta Plus XD mass spectrometer (SIO) or on a GV Micromass Isoprime continuous flow isotope ratio mass spectrometer (GV CV-IRMS) at WSU. Prior to C and N organic analyses, the inorganic C of the carbonate was removed by the addition of 2N phosphoric acid. Following acidification, capsules of faunal and C_org_ powdered rock samples were then combusted inside a Costech elemental analyzer interfaced with either the Thermo-Finnigan mass spectrometer at SIO or the GV CV-IRMS at WSU mentioned previously.

The δ^13^C_org_ and δ^15^N of particulate organic matter was analyzed from surface and bottom water collected in Niskin bottles on CTD casts. We filtered 2–4 L per sample on combusted glass fiber filters and acidified as described for fauna.

Single samples were collected from each carbonate rock for inorganic and organic C and N analyses and for δ^18^O_inorg_. Animal tissue values were averaged per rock for statistical comparisons of activity and location effects. All data were tested for normal distribution. The animal δ^13^C and δ^15^N data were normally distributed and were analyzed without transformation. The carbonate δ^13^C_inorg_, δ^13^C_org_ and δ^18^O_inorg_ values were square-root transformed prior to analysis to achieve normality.

To compare whole-assemblage trophic resource use on carbonates as a function of activity, location and habitat, community-level trophic metrics [60] were generated for isotope data using species-average values. Ranges of δ^13^C and δ^15^N, convex area in isotope space (total hull area), mean nearest neighbor distances and their SD, and mean distance to the isotope centroid were determined using programs in R by Turner et al. [61]. The isotope ranges and total hull area address trophic niche breadth. The distances to nearest neighbors and to the isotope centroid examine species packing and trophic redundancy. Standard elliptical areas (SEA) and areas corrected for sample size (SEAc) were calculated as additional measures of trophic niche breadth for each species using SIAR [62]. Differences in species metrics between active and inactive sites were examined with t tests, after log-transforming when necessary to achieve normality.

## Results

### Carbonate faunal attributes as a function of activity, location, and habitat

#### Density

Macrofaunal density on authigenic carbonates ranged from averages of 12–14 individuals 200 cm^-2^ at the shallowest sites (Jaco Summit and Quepos Landslide inactive sites) to 213 ind. 200 cm^-2^ at Mound 12 active sites. However, several rocks at Mound 12 active sites had densities > 600 ind. per 200 cm^-2^. Average densities were 2.4 times greater on Mound 11 and Mound 12 (Table 2) at active than inactive sites. The active/inactive difference was even greater at the two deepest locations, Mound Quepos (4.4 x) and Jaco Wall (7.2 x), although overall densities were lower. Overall, carbonates at active sites exhibited higher densities (180.2 ± 48.6 ind. 200 cm^-2^) than at inactive sites (33.7 ± 8.9 ind. 200 cm^-2^) (2 Way ANOVA F_1, 31_ = 9.123, P = 0.005).

**Table 2:** Summary of total macrofaunal densities on carbonate rocks at active seeps and inactive sites off Costa Rica as a function of (A) Location and (B) Habitat.

A.				
**Site**	**Activity**	**Sample Size (n)**	**No. Ind. per 200 cm^2^ Mean (SE)**	**No. Ind./m^2^**
**Quepos Landslide**	Active	0	NA	NA
**Quepos Landslide**	Inactive	5	14.3 (8.0)	712.00
**Jaco Summit**	Active	0	NA	NA
**Jaco Summit**	Inactive	2	11.5 (10.5)	577.00
**Mound 11**	Active	4	112.2 (93.9)	5609.00
**Mound 11**	Inactive	6	46.7 (34.9)	2337.00
**Mound 12**	Active	14	212.9 (125.3)	10646.00
**Mound 12**	Inactive	3	86.1 (73.8)	4305.00
**Mound Quepos**	Active	1	89.9 (NA)	4494.00
**Mound Quepos**	Inactive	2	20.2 (16.5)	1010.00
**Jaco Wall**	Active	1	84.2 (NA)	4207.00
**Jaco Wall**	Inactive	3	11.7 (9.6)	585.00
B.				
**Habitat**	**Activity**	**Sample Size (n)**	**No. Ind. per 200 cm^2^ Mean (SE)**	**No. Ind./m^2^**
**Tubeworms**	Active	5	246.4 (170.0)	12319
**Mussel Bed**	Active	9	192.2 (130.0)	9608
**Microbial Mat**	Active	4	107.1 (97.1)	5353
**Clambed**	Active	2	107.0 (92.6)	5351
**Non Seep**	Inactive	14	43.9 (33.4)	2193

In the 2-way analysis testing the effect of location and activity, location did not have a significant influence on density (P = 0.328), and there was no interaction between activity and location (P = 0.598). However, when location effects on density were tested alone via 1-way ANOVA, location had a significant effect (F_5, 31_ = 3.6, P = 0.011) with lower densities at Quepos Landslide and Jaco Summit (inactive sites only) than at Mounds 11 and 12 (with active and inactive sites) (a posteriori LSD test). The habitat effect on fauna density was also significant (2 Way ANOVA; P = 0.037). Total macrofaunal densities were higher on rocks collected from mussel beds (246 ind. 200 cm^-2^) and tubeworm aggregations (192 ind. 200 cm^-2^) than rocks collected from active sedimented habitats (clam beds and microbial mats–both 107 ind. 200 cm^-2^); density on inactive rocks was lower than on active rocks from mussel bed and tubeworm habitat at the 95% confidence level (Tukey’s HSD).

#### Composition

**Fig 5:**
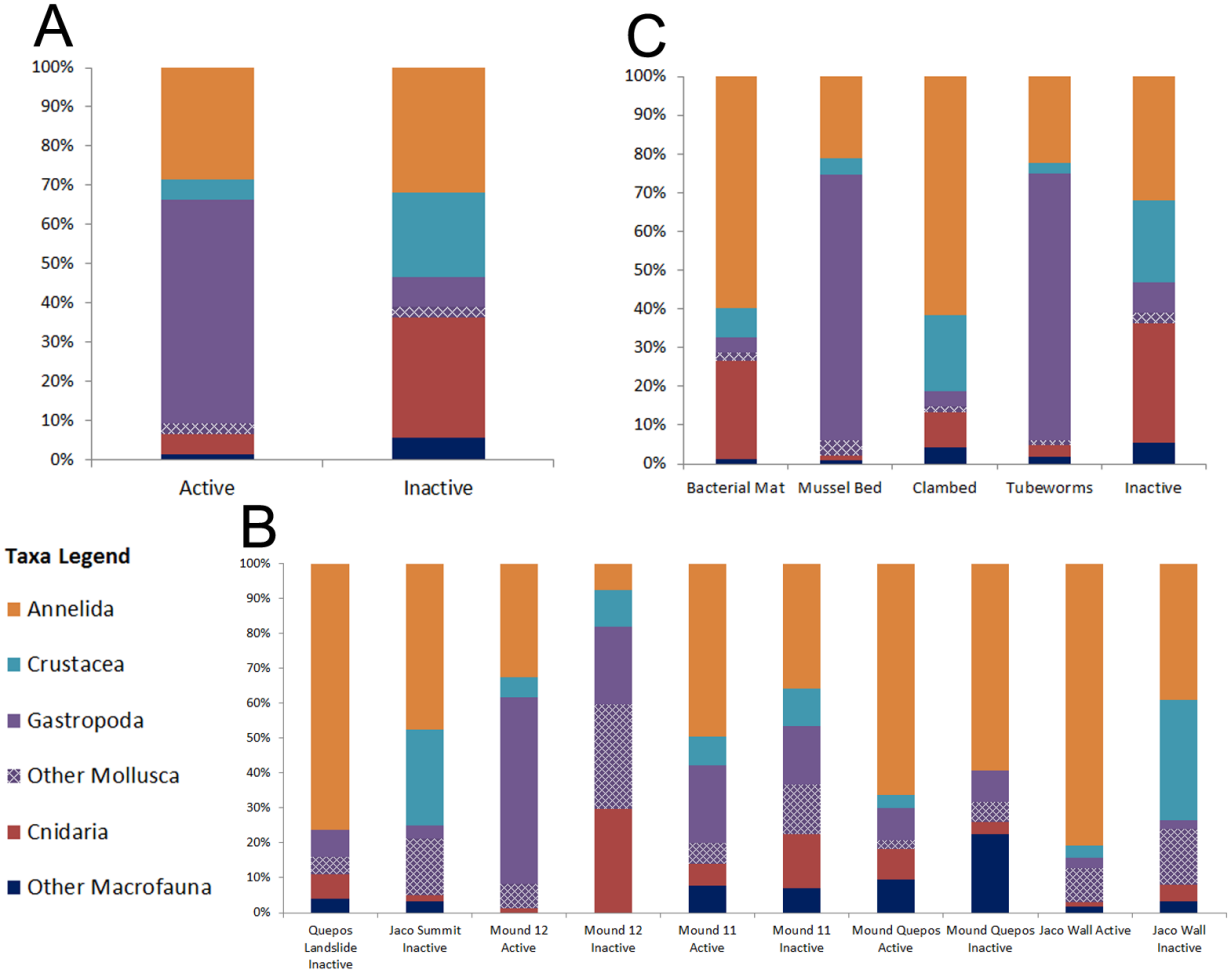
Higher taxonomic composition (percent) of metazoan macrofaunal individuals on Costa Rica margin carbonates as function of A. Activity; B. Site; C. Habitat. Values were obtained by pooling all samples.

Rocks from active vs inactive areas supported different macrofaunal assemblages (ANOSIM R = 0.416, P = 0.01) with 88% dissimilarity. Gastropods were dominant on active rocks, while crustaceans and cnidarians were better represented on inactive rocks. Polychaete representation was similar on both (Fig 5A). Location influenced faunal composition as well (ANOSIM R = 0.5, P = 0.01). Polychaete species were the top-ranked taxa at the two shallowest and two deepest locations, whereas gastropods were dominant on Mounds 11 and 12. Crustaceans comprised > 10% of the fauna at Jaco Summit, Jaco Wall and Mound 11 (Fig 5B).

The habitat matrix surrounding each rock appeared to play a significant role in determining macrofaunal assemblage composition. Significant differences were observed among the active habitats (all ≥990 m; ANOSIM R = 0.496, P = 0.002). Rocks from mussel beds and tubeworm bushes had similar assemblages, with dominance by gastropods, including various limpet and snail groups (Fig 5C). Active rocks collected from sedimented environments (clam beds and bacterial mats) also had similar faunas, with dominance by polychaetes and crustaceans. The eight most abundant species on rocks from mussel beds were gastropods (47% of total), whereas the four most abundant species on rocks from tubeworm aggregations were polychaetes (comprising 26% of the total) (Table 3). Gastropods were a minor component of the fauna on rocks in clam beds and on microbial mats (12%). The carbonate fauna on microbial mat rocks was dominated by polychaetes (a lacydonid and spionid species comprised 27% of the individuals). Clam bed carbonates exhibited the greatest taxonomic range, with annelids, crustaceans, echinoderms and cnidarians all well represented; a syllid and gammarid amphipod species formed 27% of the total. The inactive rocks from 990–1800 m depth range exhibited relatively equal proportions of annelids, gastropods and crustaceans, but also had the greatest proportion of other taxa. Macrofaunal composition on rocks from mussel beds differed from those on clam beds (ANOSIM R = 0.655, P = 0.018) and on bacterial mats (ANOSIM R = 0.541, P = 0.038), but rocks in tubeworm beds did not (ANOSIM R = 0.069, P = 0.273).

**Table 3:** Dominant macrofaunal taxa on Costa Rica seep carbonates as a function of (A) Activity (B) Location and (C) Habitat.

A.							
**ACTIVE > 900 m**	**%**	**INACTIVE > 900 m**	**%**				
*Pyropelta* sp. 2	11.02	Hydroids sp. (colonial)	26.15				
*Provanna* sp.	9.19	Gammaridae unid.	13.72				
*Neolepetopsis* n sp.	6.89	*Thrausmatos* sp.	7.56				
*Lepetodrilus guaymasensis*	6.54	Hydrozoa	7.15				
*Pyropelta corymba*	5.7	Gammaridae sp. 1	4.22				
*Pyropelta* sp. 1	4.95	Syllidae unid.	3.99				
*Provanna* cf. *laevis*	4.6	Tanaidacea	2.98				
*Chrysopetalum sp*.	4.33	*Provanna* sp.	1.66				
*Paralepetopsis* sp. a	3.71	Polynoidae unid.	1.57				
Hydroids sp. (colonial)	3.4	Phyllodocidae unid.	1.37				
B.				C.			
**QUEPOS LANDSLIDE**	**%**	**JACO SUMMIT**	**%**	**CLAMBED**	**%**	**MAT**	**%**
Sabellidae unid.	63.48	Gammaridae sp. 2	10.62	*Thrausmatos* sp.	20.93	Hydroids sp. (colonial)	25.33
Anthozoa sp.	5.93	*Thrausmatos sp*.	8.67	Gammaridae unid.	15.16	*Thrausmatos* sp.	17.72
Gastropoda unid. limpet	4.81	Gammaridae unid.	8.67	Sabellidae sp. 1	13.03	Lacydoniidae spp.	11.95
Brachiopoda	3.96	Syllidae unid.	5.77	Syllidae unid.	7.82	Syllidae unid.	5.32
Pectinoidea	3.37	Solemyidae sp.	5.07	Hydroids sp. (colonial)	6.49	Gammaridae sp. 5	5.25
*Lepetodrilus guaymasensis*	2.92	Bivalvia sp. r	4.51	Tanaidacea	3.41	Paraonidae unid.	4.63
Terebellidae unid.	2.47	Hesionidae sp. 1	3.47	Ophiuroidea unid.	2.66	Ampharetidae spp.	3.72
Bivalvia unid.	1.65	Paraonidae unid.	3.47	Cirratulidae unid.	2.52	Spionidae sp. 3	1.47
Lumbrineridae	1.32	Ampeliscid	3.47	Capitellidae unid.	2.52	Gammaridae unid.	1.26
Spionidae unid.	1.23	Isopoda unid.	3.17	Anthozoa sp.	2.39	*Lepetodrilus guaymasensis*	1.07
**MOUND 11—Active**	**%**	**MOUND 11—Inactive**	**%**	**MUSSEL BED**	**%**	**TUBE WORMS**	**%**
*Provanna* sp.	11.87	Gammaridae unid.	27.40	*Provanna* sp.	12.97	*Pyropelta* sp. 2	18.40
*Thrausmatos sp*.	9.98	Hydrozoa	15.13	*Pyropelta* sp. 2	9.84	*Provanna* sp.	8.60
Lacydoniidae spp.	8.35	Gammaridae sp. 1	9.25	*Neolepetopsis* nov. sp.	8.48	*Neolepetopsis* nov. sp.	8.24
*Paralepetopsis* sp. a	7.84	Tanaidacea	4.05	*Pyropelta corymba*	7.98	*Lepetodrilus guaymasensis*	7.96
*Pyropelta* sp. 2	7.75	Syllidae unid.	3.53	*Lepetodrilus guaymasensis*	7.50	*Galapagomystides* sp.	6.55
Gammaridae unid.	7.10	Phyllodocidae unid.	3.00	*Pyropelta* sp. 1	7.19	*Pyropelta corymba*	5.47
*Provanna* cf. *laevis*	6.09	*Provanna* sp.	2.77	*Provanna* cf. *laevis*	6.63	*Paralepetopsis* sp. a	5.26
*Pyropelta corymba*	3.49	Ophiuroidea unid.	2.51	*Paralepetopsis* sp. a	3.96	*Pyropelta* sp. 1	4.32
Syllidae unid.	3.10	Polynoidae unid.	2.31	*Kiwa puravida*	2.93	*Provanna* cf. *laevis*	3.91
Hydroids sp.	3.00	*Thrausmatos* sp.	1.82	Terebellida unid.	2.56	*Neomphalina* sp.	3.64
**MOUND 12—Active**	**%**	**MOUND 12—Inactive**	**%**				
*Pyropelta* sp. 2	12.16	Hydroids sp.	60.39				
*Provanna* sp.	9.32	*Thrausmatos sp*.	16.00				
*Neolepetopsis* nov. sp.	8.32	Syllidae unid.	3.74				
*Lepetodrilus guaymasensis*	7.59	Gammaridae unid.	2.87				
*Pyropelta corymba*	6.37	Ophiuroidea sp. 1	1.43				
*Pyropelta* sp. 1	5.86	*Polycirrus* sp. 1	0.96				
*Provanna* cf. *laevis*	4.64	Tanaidacea	0.94				
*Thrausmatos sp*.	3.73	Lumbrineridae	0.93				
Hydroids sp.	3.64	Hesionidae sp. 3 (deletabile)	0.91				
*Paralepetopsis* sp. a	3.30	Hesionidae spp.	0.83				
**MOUND QUEPOS—Active**	**%**	**MOUND QUEPOS—Inactive**	**%**				
*Galapagomystides* sp.	58.33	Actiniaria	13.98				
*Escarpia* sp.	16.67	Ophiuroidea sp. 5	11.98				
Nemertea spp.	14.29	Polynoidae unid.	7.76				
Hesionidae spp.	3.57	Ophiuroidea sp. 1	7.30				
*Diploura* sp.	1.19	*Provanna* sp.	5.99				
Cirratulidae unid.	1.19	Ophiuroidea sp. 6a	5.99				
Bivalvia juv.	1.19	*Lepetodrilus guaymasensis*	3.99				
Ampharetidae sp. 1	1.19	Ophiuroidea sp. 5a	3.99				
*Eurythoe sp*.	1.19	*Bathyacmaea* sp.	3.99				
Chiton sp. 4	1.19	Hydrozoa	3.76				
**JACO WALL—Active**	**%**	**JACO WALL—Inactive**	**%**				
Sabellidae sp. 1	33.16	Syllidae unid.	14.13				
Syllidae unid.	9.63	Hesionidae sp. blue	11.11				
Terebellidae sp. 2	8.56	Tanaidacea	10.57				
Cirratulidae unid.	6.42	Paraonidae unid.	9.77				
Capitellidae unid.	6.42	Cirripedia	9.72				
*Lepetodrilus guaymasensis*	4.28	Cirratulidae unid.	6.52				
Gammaridae unid.	3.21	Gammaridae sp. 5	6.21				
Ampharetidae spp.	3.21	Terebellida unid.	4.44				
Polynoidae unid.	2.67	Hydroids sp.	4.44				
Hesionidae spp.	2.14	Nemertea spp.	3.90				

**Fig 6:**
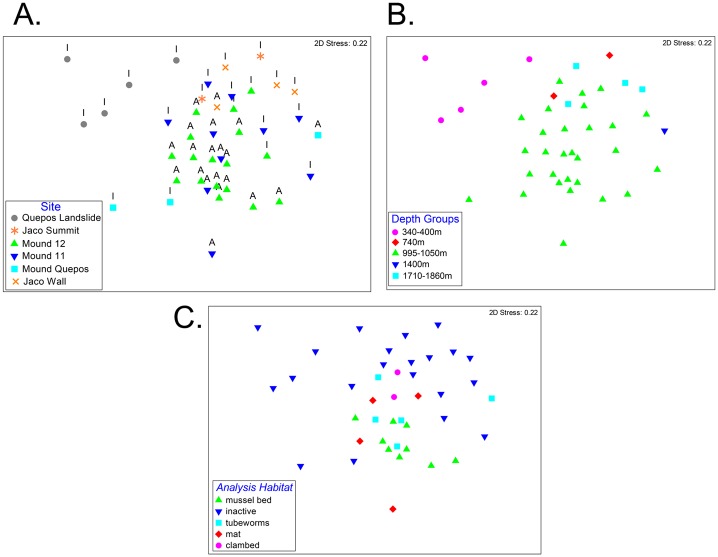
Multidimensional scaling plot of macrofaunal composition on seep carbonates represented as a function of A. Site and Activity; B. Water Depth; C. Habitat.

When carbonate faunas were evaluated as a function of water depth (ANOSIM R = 0.0453, P = 0.01), significant differences in composition were found only between the shallowest assemblages (340–400 m at Quepos Landslide) and those at 741 m (Jaco Summit) or below 1450 m (Jaco Wall; Fig 6B)

Macrofaunal community composition and its interaction with activity, habitat (Fig 6A) and location (Fig 6C) was tested with a fixed 3-factor PERMANOVA; the first run included all the rocks and then a separate test was performed with deeper (> 900m) sites only (the only depths where samples were collected from both active and inactive areas at each site). For carbonates ≥ 900 m, activity, site and habitat were found to have significant influence on composition (Table 4). The community interaction of site and activity was always significant at the 95% confidence level. On the other hand, activity and habitat did not appear to significantly interact. In addition, the carbonate rock communities differ in composition at all three levels (activity, site and habitat; PERMANOVA results in Table 4).

**Table 4:** PERMANOVA analysis to examine effects of habitat, site and activity (and their interaction) on composition of macrofauna on carbonate rocks at Costa Rica seeps. Comparisons were made for all rocks at all sites (upper) and for those at site > 900 m (lower), where both active and inactive areas co-occurred.

**ALL ROCKS**					
Source	df	SS	MS	Pseudo-F	P
Habitat	4	17078	4269.6	1.48	0.01
Site	4	28898	7224.6	2.50	0.00
Habitat * Site	5	15996	3199.2	1.11	0.23
Residuals	27	78121	2893.4		
Site	5	33362	6672.5	2.30	0.00
Activity	1	4949.6	4949.6	1.70	0.02
Site * Activity	3	12232	4077.3	1.40	0.02
Residuals	31	90059	2905.1		
**>900m**					
Source	df	SS	MS	Pseudo-F	P
Habitat	4	17290	4322.5	1.53	0.00
Site	3	16211	5403.7	1.92	0.00
Habitat * Site	5	16085	3217	1.14	0.18
Residuals	21	59241	2821		
Site	3	16570	5523.2	1.90	0.00
Activity	1	4949.6	4949.6	1.71	0.02
Site * Activity	3	12232	4077.3	1.41	0.01
Residuals	26	75388	2899.5		

#### Diversity

**Fig 7:**
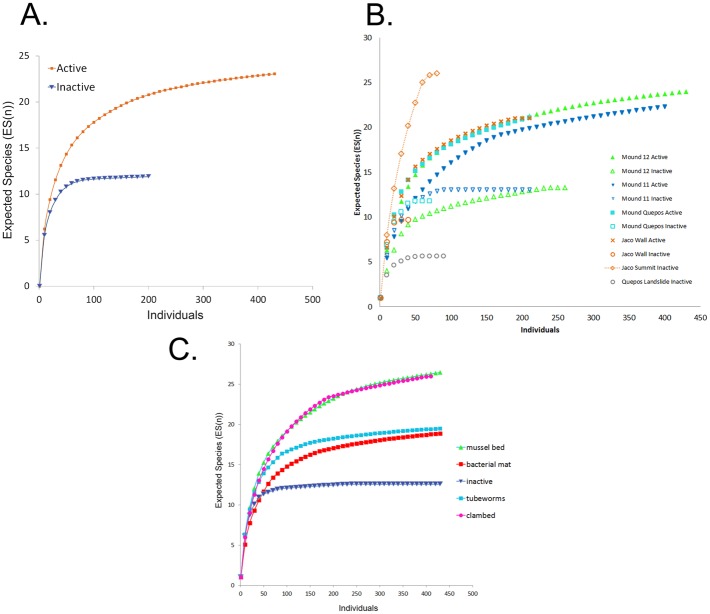
Rarefaction curves depicting macrofaunal diversity on seep carbonates as a function of A. Activity; B. Site; C. Habitat.

Macrofaunal taxon richness was higher on active carbonates (26.4 ± 2.5 species) than inactive carbonates (12.5 ± 1.5 species) (Table 5; Fig 7A) (ANOVA F_1, 31_ = 6.51, P = 0.016). Location also had a significant effect on species richness (F_5, 31_ = 3.625, P = 0.011) but did not interact with activity. The shallowest locations, both with relatively low oxygen and inactive habitats only, exhibited the highest (Jaco Summit) and the lowest (Quepos Landslide) diversities in terms of taxon richness and rarefaction on carbonates (Fig 7B). Mound 11 and Mound Quepos seep diversities were similar, Mound 12 was slightly higher and Jaco Wall slightly lower (Fig 7B). These results suggest that within the studied range (400–1850 m), water depth does not regulate diversity patterns of seep carbonate assemblages. Taxon richness also varied with habitat (ANOVA F_4, 36_ = 5.38, P = 0.002). Rocks near tubeworm bushes and microbial mats had lower rarefaction diversities than those in mussel and clam bed habitats, and inactive rocks exhibited the lowest rarefaction diversity (Fig 7C).

**Table 5:** Diversity indices for carbonate macrofauna at Costa Rica seep.

**A.**												
**Activity**	**Active**		**Inactive**									
**Function**	**mean**	**SE**	**mean**	**SE**								
**n**	**26**		**24**									
**S**	24.4	2.3	12.0	1.4								
**N**	319.2	71.5	48.8	9.9								
**d**	4.4	0.3	3.0	0.3								
**J'**	0.8	0.0	0.8	0.0								
**Fisher**	8.4	0.7	7.8	1.2								
**H'(loge)**	2.3	0.1	1.8	0.2								
**H'(log10)**	1.0	0.0	0.8	0.1								
**1-Lambda'**	0.8	0.0	0.7	0.1								
**B.**												
**Location**	**Quepos Landslide**		**Jaco Summit**		**Mound 12**		**Mound 11**		**Mound Quepos**		**Jaco Wall**	
**function**	**mean**	**SE**	**mean**	**SE**	**mean**	**SE**	**mean**	**SE**	**mean**	**SE**	**mean**	**SE**
**n**	**5**		**2**		**17**		**10**		**3**		**4**	
**S**	5.6	1.6	26.0	4.0	23.3	2.7	17.8	3.2	16.5	4.3	14.2	3.6
**d**	1.4	0.4	5.9	1.1	4.2	0.3	3.6	0.4	4.0	0.7	3.5	0.4
**J'**	0.6	0.1	0.9	0.0	0.7	0.0	0.7	0.0	0.9	0.0	0.9	0.0
**Fisher**	2.4	0.8	16.7	6.2	7.8	0.8	6.7	0.9	11.2	2.0	9.5	1.8
**H'(loge)**	1.0	0.3	2.9	0.1	2.2	0.1	2.0	0.1	2.3	0.2	2.3	0.1
**H'(log10)**	0.4	0.1	1.3	0.1	0.9	0.1	0.9	0.1	1.0	0.1	1.0	0.0
**1-Lambda'**	0.5	0.1	0.9	0.0	0.8	0.0	0.8	0.0	0.9	0.0	0.9	0.0

#### Environment-Fauna Relationships

A combination of environmental (depth, temperature, oxygen concentration) and isotope data (δ^13^C_org_, δ^13^C_inorg_ and δ^18^O) yielded the strongest relationship between environmental variables and community data (RELATE test, Rho = 0.420), whereas the inclusion of habitat and site as environmental variables did not yield a better fit (Rho = 0.275). Model explanatory power was better for the inactive rocks (Rho = 0.484) compared to the active rocks (Rho = 0.305).

**Fig 8:**
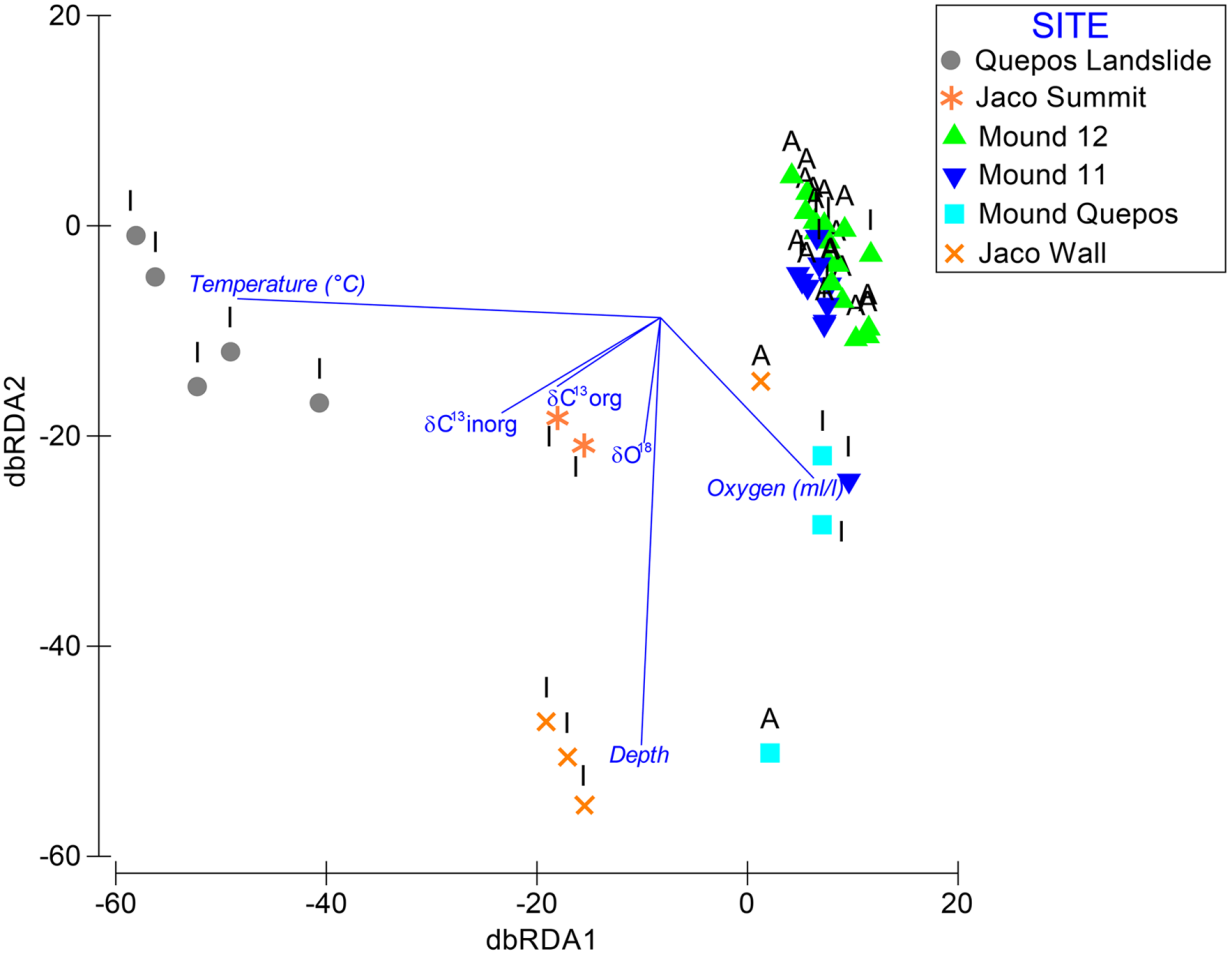
Distance based redundancy analysis (dbRDA) plot showing influence of carbonate isotopes, and temperature depth and oxygen on carbonate macrofaunal composition on rocks from different sites and activity where A = Active and I = Inactive.

For the carbonate macrofauna at active sites, DISTLM analysis revealed no significant explanatory power of environmental/hydrographic variables. However, 28% of faunal variability at inactive areas was explained by depth, temperature, oxygen, and carbonate isotope signatures as environmental proxies and all the variables were significant at the 0.05 level. The influence of the environmental variables on the inactive rocks was very high compared to the active rocks, but it was also clear that site treatment had the highest effect at the community level (Fig 8).

#### Comparison to infaunal assemblages

**Fig 9:**
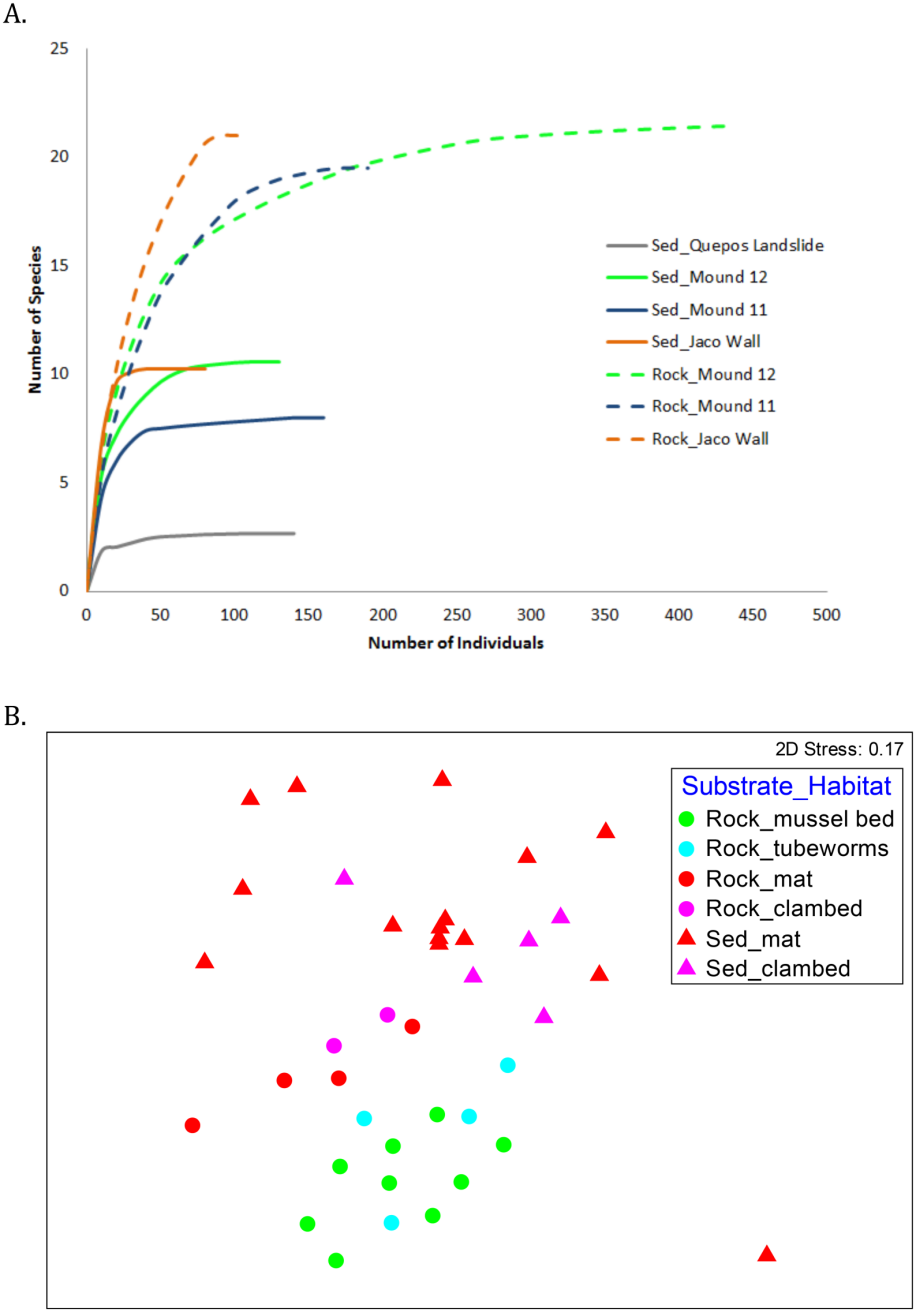
**Sediment vs Carbonate communities on the Costa Rica Margin**: A. Rarefaction curves illustrating diversity of macrofauna as a function of site and substrate; B. MDS Plot illustrating macrofaunal composition as a function of habitat and substrate.

For faunas in active seepage areas, substrate (carbonate vs sediment) appears to exert a greater influence on macrofaunal composition and diversity than does habitat or location (Fig 9). Overall faunal densities were not significantly higher in sediments (309.0 ± 55.0 ind. 200 cm^-2^) than on carbonates (184.9 ± 43.6 ind. 200 cm^-2^) (F_1, 37_ = 3.1, P = 0.088). Still, microbial mat-covered sediments contained a total macrofaunal count four times higher than that found on carbonates recovered from microbial mat habitats (437 vs 107 ind. 200 cm^-2^). Rarefaction curves (Fig 9A) and expected rarefaction taxon richness (ES_[20]_), were generally higher for carbonate fauna (Table 5) than for sediment fauna. For example, ES_[20]_ of the carbonates vs sediments was 9.2 vs 7.2 on Mound 12, 8.2 vs 6.0 on Mound 11, and 10.3 vs 9.6 on Jaco Wall. In sedimentary sulfidic mat habitats ES_[20]_ = 4.46, nearly half that of the mat and clam bed carbonate faunas (ES_[20] =_ 8.30-8.99).

Sediment (infaunal) assemblages differed from carbonate assemblages for the entire Costa Rica margin samples set (ANOSIM R = 0.556, P = 0.01), for clam bed assemblages (R = 0.636, P = 0.048) and marginally for microbial mat assemblages (R = 0.287, P = 0.063). Notably, macrofaunal assemblages on carbonates collected from mat and clam bed habitats were more similar to the infaunal assemblages than to those from mussel bed and tubeworm habitats (Fig 9B). When comparisons were made as a function of location, strong carbonate-sediment assemblage composition differences were observed at Mound 11 (ANOSIM R = 0.741, P = 0.029), and Mound 12 (R = 0.774, P = 0.001), but not at Jaco Wall (R = 0.417, P = 0.40). The sediment mat and clam bed composition did not differ (ANOSIM R = 0.089, P = 0.247). Substrate differences were generally attributed to greater representation of ampharetid, dorvilleid, hesionid, cirratulid and lacydonid polychaetes in sediments and gastropods, as well as syllid, chrysopetalid and polynoid polychaetes on carbonates [SIMPER].

### Stable Isotope Signatures

Stable isotope signatures (δ^13^C and δ^15^N) were examined in carbonate and animal tissues (from carbonates) to examine location-specific environmental variation, effects of seepage activity, relationships between the fauna and the carbonates they inhabit, and controls on trophic structure.

#### Carbonates and POM

**Fig 10:**
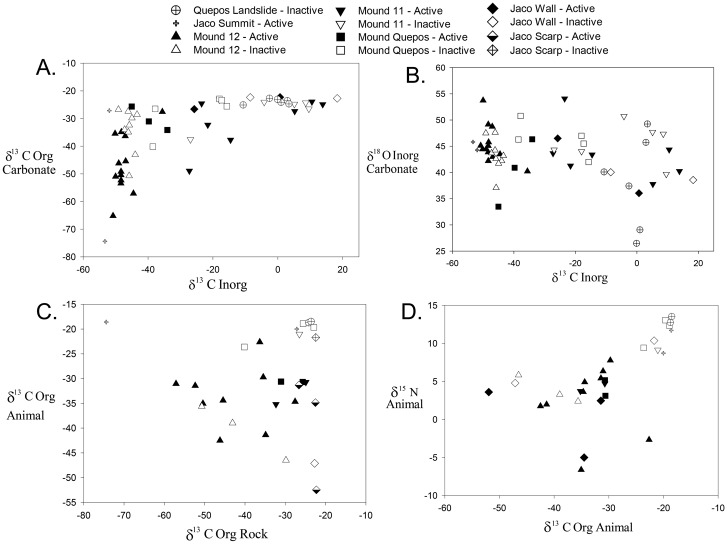
**Dual isotope plots** of A. Authigenic carbonates; B. Inorganic δ^18^O and δ^13^C for individual rocks; C; Average animal δ^13^C signatures as a function of the δ^13^Corg value of the rock they were collected on; (D) Macrofaunal δ^13^C and δ^15^N values. For A, B, and C each point represents values for a separate rock.

Authigenic carbonate δ^13^C signatures varied widely. Carbonate δ^13^C_inorg_ values were on average -26.98‰ but varied from -53.3‰ to +10.0‰. Carbonate δ^13^C_org_ exhibited an average δ^13^C value of -33.83‰, and was significantly lighter than δ^13^C_inorg_ (paired-t_54_ = 2.915, P = 0.0052). δ^13^C_org_ of carbonates ranged from -74.4 to -20.6‰. Carbonates from active sites had δ^13^C values that were significantly lighter by 10‰ for δ^13^C_org_ (t_57_ = 3.472, P = 0.001), and lighter by 14‰ for δ^13^C_inorg_ (t_53_ = 2.497, P = 0.016). Carbonate δ^13^C_org_ and δ^13^C_inorg_ signatures were significantly heavier at Quepos Landslide, Jaco Wall, and Mound 11 than at Mound 12 and Jaco Summit (C_org_: F_5, 58_ = 8.0093, P<0.001; C_inorg_: F_5,54_ = 39.525, P<0.001). There was a positive relationship between δ^13^C_inorg_ and δ^13^C_org_ (P<0.001, R^2^ = 0.44 for polynomial, and R^2^ = 0.39 for a linear fit; Fig 10A).

Average δ^13^C_org_ for particulate organic carbon was -21.6 ± 0.96‰ for surface waters (n = 8) and -20.8 ± 0.33‰ (n = 10) for POC just above the seabed (400–1800 m). Average δ^15^N values for particulate organic matter were 4.3 ± 0.6‰ at the surface and 6.9 ± 0.6‰ just above the seabed.

#### Animals

Animals collected on authigenic carbonates had wide ranging C and N stable isotopic signatures. Average δ^13^C and δ^15^N invertebrate values per rock were -31.0‰ (range -18.5 to -46.5‰) and 5.7‰ (range -4.5 to +13.4‰), respectively. The range of individual values was much greater than for rock averages. For example, the lightest animal collected had a δ^13^C of -101.5‰; this was a dorvilleid polychaete (*Dorvillea* sp.) known to derive its C from archaeal lipids [63]. The heaviest individual was a phyllodocid polychaete (*Galapagomystides* sp.) (δ^13^C = -15.8). For N the lightest animal was a bathymodiolin mussel (δ^15^N = -12.4) and the heaviest was another dorvilleid species (δ^15^N = 19.2 ‰). Average δ^13^C_org_ values of carbonate and macrofauna on a given rock were not significantly different (paired-t_18_ = 1.682, P = 0.1098), suggesting the potential for trophic linkages. δ^13^C of animal tissues did not differ for those on carbonates from active (-31.3‰) vs inactive (-30.5‰) sites, but there were significant differences among locations (F_5, 18_ = 4.39, P = 0.015), with Mound 12 faunal δ^13^C signatures being lighter than those on Jaco Summit. δ^15^N of animals also did not differ as a function of activity (active δ^15^N = 4.6‰ vs inactive 7.7‰; t_17_ = 1.474, P = 0.134), but they exhibited heavier values at Quepos Landslide, Jaco Summit and Mound Quepos than at Jaco Wall. δ^13^C_org_ values of carbonates were uncorrelated with carbonate δ^13^C_inorg_ values (P = 0.164) (Fig 10C), but were positively correlated with δ^15^N of animal tissues (R^2^ = 0.544, P = 0.003) (Fig 10D).

**Fig 11:**
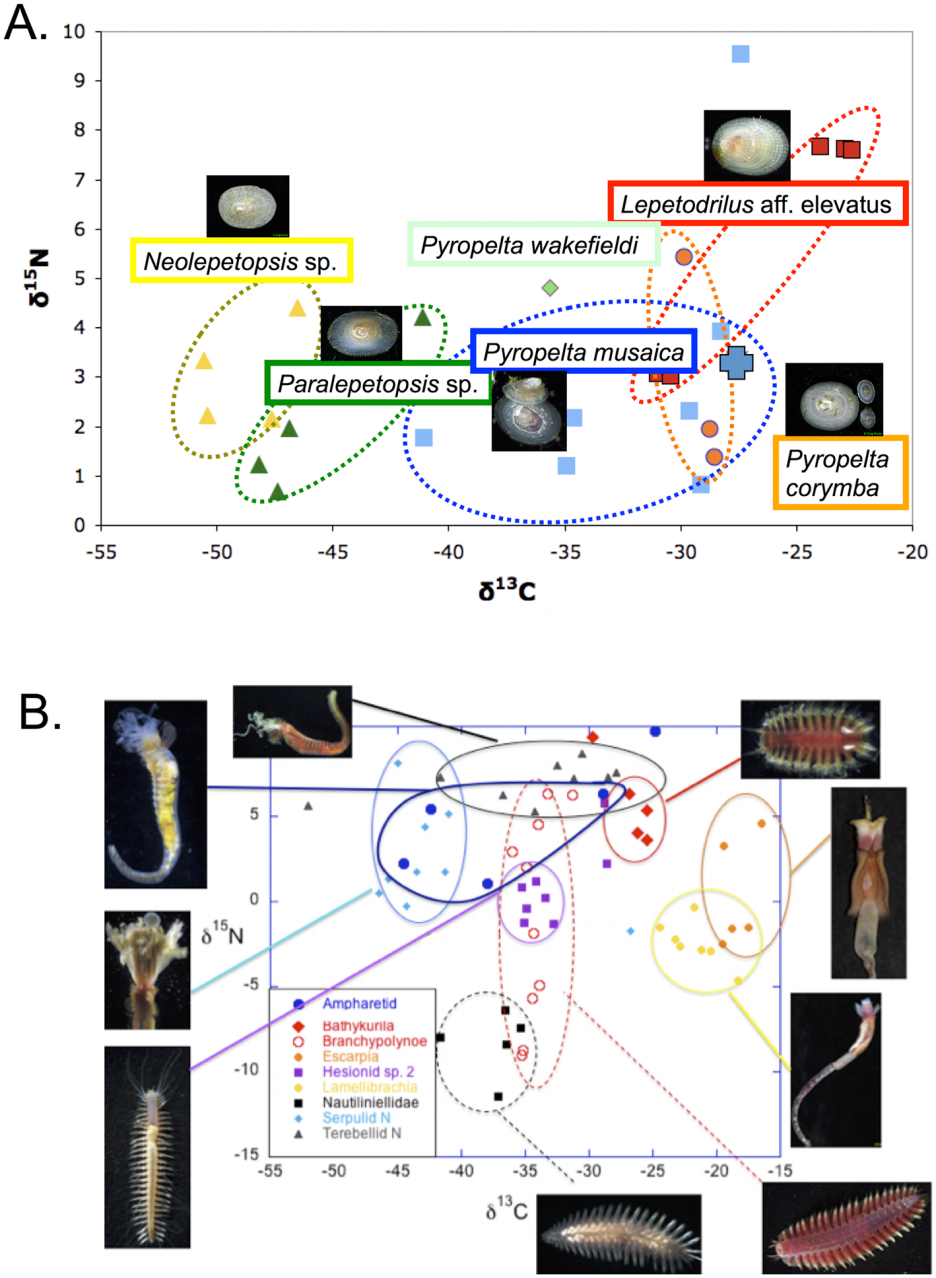
A. Dual isotope plot for polychaetes collected from carbonates on Mound 11 and 12 during AT 15–44. Costa Rica; B. Dual isotope plot for six limpet species collected on a single rock on Mound 12. In both figures each point represents a single individual.

Animals with δ^13^C signatures routinely < -50‰ included those likely to graze the carbonates, such as neolepetopsid limpets and chitons. However carbonate faunas clearly had varied diets. Within major taxonomic guilds (e.g., gastropods, polychaetes), species coexisting on the same rock often exhibited distinct average isotopic signatures, offering evidence of resource partitioning. Carbonate polychaetes exhibit a range of distinct isotopic signatures on Mounds 11 and 12 (Fig 11A) and gastropod species exhibit nutritional resource partitioning even on a single carbonate rock (Fig 11B).

#### Community Isotope Metrics

Community isotope metrics were calculated to examine the range of food sources (range δ^13^C), the number of trophic levels (range δ^15^N), overall trophic diversity (Total Hull Area, SEA, and SEAc), and species packing (distance to centroid, mean nearest neighbor distance) for carbonate rock macrofaunal assemblages. These metrics were examined as a function of activity, location, and habitat (Table 6). None of the metrics varied significantly (alpha = 0.05) between assemblages on carbonate rocks from active and inactive sites, however at the 0.10 level, mean δ^15^N range was lower (4.6 vs 9.1; t_19_ = -1.803, P = 0.087) and mean SEAc was higher (71.1 vs 15.7; t_19_ = 1.727, P = 0.100) for the active than inactive rocks (Table 6). Location influenced only δ^13^C range (Mound 12 > Jaco Wall; F_4, 16_ = 3.640, P = 0.027) and δ^15^N range (Mound 12 > Mound Quepos and Jaco Wall; F_4, 16_ = 2.900, P = 0.056), but not the metrics related to species packing or overall trophic diversity. In addition, none of the metrics examined varied among the active habitats (bacterial mat, mussel bed or tubeworm bushes) or Jaco Wall rocks (Table 6).

**Table 6:** Community isotope metrics for carbonate faunas on the Costa Rica Margin.

			Mean Distance to Centroid		Mean Nearest Neighbor		Mean Range d13C		Mean Range d15N		SEA		SEAc		Total Hull Area	
Activity		Number	mean	SE	mean	SE	mean	SE	mean	SE	mean	SE	mean	SE	mean	SE
	**Active**	**16**	5.5	0.9	3.4	0.8	-30.5	1.8	4.6	1.2	61.1	15.8	71.1	19.2	148.7	51.9
	**Inactive**	**5**	6.0	1.5	3.0	0.4	-25.1	3.4	9.1	1.9	51.4	30.3	15.7	9.6	36.2	21.9
**Site**																
	**Jaco Scarp**	**3**	1.7	0.8	1.3	0.6	-19.0	0.5	11.3	1.4	9.4	6.4	11.9	7.9	10.7	7.6
	**Mound 11**	**3**	6.4	1.9	2.5	0.3	-29.3	4.8	5.6	1.9	70.9	31.8	77.3	33.9	132.2	52.7
	**Mound 12**	**10**	6.3	1.1	4.1	1.0	-34.0	1.7	2.8	1.4	69.4	19.0	72.8	24.8	179.4	81.1
	**Mound Quepos**	**4**	5.3	2.1	3.5	1.6	-25.1	3.2	8.6	2.6	62.7	52.2	55.3	46.6	98.7	84.3
	**Quepos Landslide**	**1**	9.9		3.1		-28.8		6.4							
**Active Habitat**																
	**Bacterial Mat**	**3**	6.4	2.0	3.9	1.7	-32.6	2.1	6.2	1.7	55.8	39.3	63.7	40.3	96.8	71.4
	**Mussel Bed**	**8**	5.3	1.3	3.3	1.3	-32.9	2.5	3.6	1.9	59.1	20.7	71.1	29.1	139.8	67.7
	**Tubeworms**	**3**	7.3	3.0	4.4	1.9	-29.7	3.8	2.1	2.4	102.7	50.9	114.2	56.8	310.1	191.9
	**Jaco Rocks**	**2**	2.4	0.5	1.8	0.5	-19.3	0.7	10.2	1.5	14.0	7.6	17.7	9.2	16.1	9.3

The above analyses were carried out at the individual rock level, with calculations made for the fauna on each carbonate rock and then averaged. This analysis yields greater trophic diversity on active than inactive rocks. However, when all data are combined and average species isotope signatures are determined for all inactive and all active rocks, metrics of trophic diversity (SEAc and Total Hull area) appear somewhat larger for inactive carbonates.

## Discussion

### Agents structuring carbonate communities

Carbonate faunas in the deep ocean are an overlooked and understudied source of macrofaunal biodiversity. At methane seeps carbonates represent an abundant, porous substrate with high organic content that provides habitat, refuge and food. In many ways, the assemblages resemble those of shallow water rocky shorelines and jetties, with dominance by snails, limpets, mussels, amphipods, and polychaetes. However, in seep systems the algal grazers common in shallow water are replaced by microbial grazers, and the filtering mussels and reef-building polychaetes are replaced by symbiont-bearing mussels (that can also filter feed) and siboglinid polychaetes (tubeworms). Recent studies show that authigenic seep carbonates contain diverse microbial assemblages of archaea and bacteria. Archaeal assemblage composition (ANMEs) in particular is more sensitive to seepage level, whereas bacteria appear to be more dependent on substrate type [64]. However, even on carbonates that appear inactive (i.e., don’t support seep megafauna), there are ANME Archaea capable of anaerobic methanotrophy [15].

Seep carbonate macrofaunal assemblages are distinct from those in nearby soft-substrate seep sediments, which are better studied [30]. The carbonate communities are influenced strongly by presence of seepage activity and by the surrounding habitat matrix. Rocks at active sites support a gastropod-dominated assemblage distinct from those on rocks at inactive sites. Carbonates associated with biogenic habitats (mussels/tubeworms) support faunas different from carbonates in a sediment matrix (clam bed or microbial mat habitats; Fig 7B), also with greater gastropod representation. Possibly the clam bed and mat sediments preclude migration and colonization of gastropods or release sulfides toxic to selected species.

Several studies have examined macrofauna of carbonate crusts in other seeps at depths comparable to those studied off Costa Rica. Average faunal densities observed here for Costa Rica margin carbonates at active sites were 3-20x higher (e.g., 192–246 ind. 200 cm^-2^) than those reported for carbonates from seeps at the Amon Mud Volcano and Pockmark areas of the Nile Deep-sea Fan (NDSF) (1000–1700 m; 250 micron mesh size; [39]) and 30x higher than for carbonates from the Del Mar Seep in the NE Pacific at 1020 m despite some shared gastropod taxa [41]. However the Costa Rica carbonate densities were slightly lower than those reported from the NE central Basin of the Marmara Sea (306 ind. 200 cm^-2^) at 1111 m [1]. Carbonates from inactive sites on the Costa Rica margin at 400-700m had mean densities of 12–14 ind. 200 cm^-2^, which were comparable to those reported by Ritt et al. [40] from a 1000 m reference (inactive) site (15 ind. 200 cm^-2^) in the NDSF. Gastropods or mussels were dominant on the carbonate rocks studied in the Marmara Sea [1], at the Del Mar Seep [41] and on the rocks at Pockmark in the NDSF [40]. At Amon Mud Volcano, cnidarians were dominant [40] which was similar to our findings off Costa Rica where hydroids were common on inactive carbonates. Shannon-Wiener diversities of the Costa Rica active carbonate faunas (H’_loge_ = 2.13–2.57) were, generally higher than those reported for the Amon Mud Volcano (0.96) or Pockmark area (2.08) [40] but similar to those from the Del Mar Seep (2.59; [41]). Evenness (J’) was comparable in the NDSF (0.60–0.79), Del Mar Seep (0.75) and off Costa Rica (0.64–0.75) for active carbonates. Many more mollusk species were observed on carbonates off Costa Rica than at the other sites (A. Waren unpublished observation). It is unclear whether this is due to high diversity in the region or to the much larger number of rocks examined in this study (n = 38) than in the other 3 studies discussed here (n = 3 to 6).

Our observation of 1.5 to 2 times higher macrofaunal diversity (based on rarefaction estimates) in carbonates than in seep sediments was consistent with observations by Ritt et al. [40] at the Pockmark site and Grupe et al. [41] in the most active Del Mar seep sediments, but not the Amon Mud Volcano in the NDSF. In the Gulf of Cadiz caenogastropods are more diverse on soft than hard substrates but vetigastropods and heterobranchia did not show substrate–related diversity patterns [65]. Seep sediment macrofaunal assemblages are heavily dominated by polychaetes [31], whereas carbonates appear to support many gastropod (coiled snail and limpet faunas) and polychaete species along with numerous other attached (e.g., cnidarians), grazing (chitons), filter feeding (bathymodiolin mussels, echinoderms), and predatory forms (amphipods, galatheids, kiwas) (Table 3) [1, 40, 41].

While depth patterns are well studied for soft-sediment macrofauna in the deep sea [66], few studies have examined the influence of depth or hydrography on the diversity of hard substrates in the deep sea. The carbonate faunas do not exhibit a mid slope diversity maximum exhibited by many soft sediment transects [67]. The macrofauna of mud volcanos in the Gulf of Cadiz (NE Atlantic), sampled from 200 to 4000 m also did not exhibit a midslope diversity maximum [68]. Off Costa Rica the intense OMZ at 400 m (Fig 2) yields exceptionally low diversity at Quepos Landslide on carbonates (Fig 7b) and in sediments (Fig 9), where oxygen concentration was 0.04 ml l^-1^. Surprisingly, the highest diversity occurs on inactive carbonates at Jaco Summit just a few hundred meters deeper.

### Comparison to other hard-substrate ecosystems

Since the advent of quantitative core sampling global comparisons of densities and diversities across environments or geographic regions have become routine for deep-sea sediment communities, but quantitative macrofaunal data for hard-substrate biota in the deep sea are less readily accessible. Reviewing information for other reducing ecosystems (Table 7) we find that total macrofaunal densities on active carbonate substrates off Costa Rica are similar to those on whale skeletons at 960–1910 m (123–328 ind. 200 cm^-2^) off southern California [69], and slightly lower than those colonizing wood at the Håkon Mosby Mud Volcano (300 ind. 200 cm^-2^) [42]. Considering non-reducing systems in the deep sea, we find that densities on inactive carbonates outside the OMZ (44 ind. 200 cm^-2^) are much higher than those on manganese nodules in the abyssal Pacific (22 ind. 200 cm^-2^) [70], seamounts off California (1–2 ind. m^-2^) [71], and background rocks at 960 m off San Nicholas Island (10 ind. 200 cm^-2^) [69], but lower than epibenthic fauna on sponge stalks at abyssal station M (350 ind. 200 cm^-2^) in the E. Pacific [72]. The inactive carbonates within the OMZ have notably lower densities (11–14 ind. 200 cm^-2^).

**Table 7:** Macrofaunal densities on hard susbtrates in the deep sea and shallow waters.

Substrate	Location	Water Depth (m)	Latitude/Longitude	Density/unit area	Density ind./200cm^2^	# individuals	# species	Surface area	Dominant taxa	Reference
Manganese nodules	equatorial and central North Pacific	4500–5800	5°N, 125°W 30°N, 157°W	1090 ind./m^2^	21.8	120	32	0.11 m^2^		Mullineaux (1987)
Whale skeleton	San Nicolas	960	33°20'N, 119°59'W	6169 ind./m^2^	123.38	5120	190	0.83 m^2^	Bivalvia	Baco and Smith (2003)
Whale skeleton	San Catalina Basin	1240	33°12'N, 118°29'W	16375 ind./m^2^	327.5	20632	180	1.26 m^2^	Bivalvia	Baco and Smith (2003)
Whale skeleton	San Clemente Basin	1910	32°26'N, 118°9'W	11005 ind./m^2^	220.1	11555	102	1.05 m^2^	Bivalvia	Baco and Smith (2003)
Vent Mussel Beds	Mid-Atlantic Ridge	1600	37°17'N, 32°16'W	811 ind./L of mussel		20044	25	24.7 L of mussel	Crustacea	Van Dover and Trask (1999)
Deep-sea rocks	San Nicolas	960	33°15'N, 119°56'W	490 ind./m^2^	9.8	147	26	0.3 m^2^		Baco and Smith (2003)
Seamount	Davidson	1246–3289	35°43'N 122°43'W	0.87 ind./m^2^	0.0174	59933	148		Cnidaria	Lundsten *et al*. (2009)
Seamount	Pioneer	811–1815	37°21'N, 123°26'W	2.19 ind./m^2^	0.0438	36430	110		Cnidaria	Lundsten *et al*. (2009)
Seamount	Rodriguez	619–2120	34°01'N, 121°04'W			38087	133		Echinodermata	Lundsten *et al*. (2009)
Sponge stalks	Station M	4100	34°45'N, 123°00'W	17572 ind./m^2^	351.44	1933	104	0.11 m^2^	Polychaeta	Beaulieu (2001)
Wood	Haakon Mosby Mud volcano	1257	72°00'N, 14°43'E	14988 ind./dm^3^	299.76	2398			Bivalvia	Gaudron *et al*. (2010)
Rocky Shore	Australia-Tropical (exposed/sheltered)	intertidal	23o S 151o E	97.6/ 31.5 per 400 cm^2^	49/16		12/14.8	400 cm^2^	Cirripedia	73
Rocky Shore	New Zealand—Temperate (exposed/sheltered)	Intertidal	45o S 170o E	265/64.8 per 400 cm^2^	133/33		12/15.2	400 cm^2^	Cirripedia	73
Mussel beds	Eagle Island Alaska	0	54°62′N, 159°99′W	970 ind./L of mussel		78353	70	80.7 L of mussel	Polychaeta	Van Dover and Trask (1999)
Rocky shore	South-Central California (early/mid/late succession)	intertidal algal mats on boulders	34o 25'N 119o 41'W	78/316/294 per 0.01m^2^	156/632/588		214	0.09 m^2^	Crustacea/ Polychaeta	75

At a coarse taxonomic level the composition of carbonate faunas at active Costa Rica seep sites (Fig 4), bears remarkable resemblance to the biota of temperate and tropical rocky intertidal shorelines (Table 7; [73,74,75]), with the exception of barnacles, which are often space-dominant in the latter. Bacteria attached to carbonates appear to support high densities of grazing coiled snails and limpets, just as microalgae (and cyanobacteria) do on rocky shores. Macrofaunal densities reported for carbonates at active habitats (107–246 ind. 200 cm^-2^) are comparable to or exceed those for exposed temperate rocky shores in New Zealand (133 ind. 200 cm^-2^; [76]), while those of inactive Costa Rica carbonates outside the OMZ (44 ind. 200 cm^-2^) are comparable to those in exposed temperate rocky shores of Australia (49 ind. 200 cm^-2^; Table 7; [76]). Inside the OMZ, the Costa Rica carbonate densities (11–14 ind. 200 cm^-2^) resemble those of Australian tropical sheltered shores (15.7 ind. 200 cm^-2^; [73]). A comparison to macrofauna of subtidal reefs (Table 7) suggests that the densities at active Costa Rica carbonates are about half those of urchin barrens (498 ind. 200 cm^-2^) but only 1/10 to 1/20 those of vegetated reef habitats [76]. However taxonomic similarities are evident; as on Costa Rica carbonates, gammarid amphipods, polychaetes and gastropods are among the most numerous subtidal epifauna [76].

We can consider how authigenic seep carbonate faunas might compare to shallow coral reef faunas (Table 7). Subtidal carbonate rubble in Hawaii, with particles 2–64 mm, support higher densities of fauna (455 ind. 200 cm^-2^, 0.5 mm mesh) compared to nearby shallow sands (173 ind. 200 cm^-2^), and about twice the density of the most active Costa Rica seep carbonates (tubeworm habitat carbonates 246 ind. 200 cm^-2^; [75]). The shallow carbonate rubble exhibited high densities of amphipods and polychaetes (glycerids, nereids, capitellids, and syllids; [77]), taxa also common on seep carbonates. While comparable quantitative data (per unit surface area) are not available for macrofauna associated with carbonate derived from deep-water corals, it is clear that dead coral skeletons and coral rubble are complex substrates that support exceptionally high biodiversity [78].

### Nutrition of authigenic carbonate biota

Isotope signatures of the Costa Rica carbonates provide clear indication that AOM has played a role in generating the rocks. The rocks are on average highly depleted in δ^13^C and enriched in δ^18^O ([10]; this study), but exhibit a broad range of signatures. Those with light δ^13^C_inorg_ are believed to have formed under more intense, focused seepage [9]. In our samples those carbonates with δ^13^C_inorg_ < 40‰ exhibit a surprisingly broad range of δ^13^Corg values (-25 to -75‰), whereas rocks with heavy δ^13^C_inorg_, thought to have formed in diffuse flow, have much heavier δ^13^C_org_ (-30 to -25‰) (Fig 10a). We did not find a strong relationship between average rock δ^13^C_org_ and animal δ^13^C_org_ (Fig 8c), or an effect of local seepage activity on these values.

While we expected to see isotopically light animals under conditions of active seepage, very light δ^13^C_org_ signatures (< -50‰) were also observed in the neolepetopsid limpets and chitons collected on carbonates at inactive sites, indicating that they obtain significant amounts of methane-derived carbon long after obvious signs of seepage disappear. Direct consumption of the carbonate itself may be responsible; viable archaea are abundant within inactive carbonates [15; 64] and may be one source of isotopically light organic matter. This is consistent with routine observation of carbonate fragments inside the guts of neolepetopsid limpets at seeps (A. Waren unpublished observations).

The macrofauna on carbonates may derive their organic carbon from surface-derived organic matter, sulfide-oxidizing bacteria, sulfate-reducing bacteria, aerobic methane oxidizing bacteria and anaerobic methane oxidizing archaea. On carbonates at active sites, as many as nine grazing gastropod species and a chiton can co-occur on a single rock; their distinct isotope signatures suggest they are actively partitioning microbial food resources (Fig 11b). The polychaete taxa on carbonates also exhibit a broad range of isotopic signatures reflecting diverse feeding modes that range from symbiosis to bacterial grazing, filter feeding, deposit feeding, carnivory and archivory (the dependence on archaeal-derived carbon) (Fig 11a). Similar partitioning of microbial food resources was observed for multiple polychaete species within the family Dorvilleidae in methane seep sediments off OR and CA [79], and for lepetopsid and lepetodrilid limpets at hydrothermal vents on the east Pacific Rise [80].

Clearly, both active and inactive carbonates support macrofaunal assemblages with diverse trophic pathways. We observe a greater diversity of food resources within any single rock at active sites (alpha trophic diversity, Table 6), but a greater spread of trophic resources across inactive rocks (beta trophic diversity), yielding high overall trophic diversity among seep macrofauna. This appears to be due to the occurrence of both photosynthetic and chemosynthetic nutritional pathways, and to the broad range of microbial food sources available in and on authigenic carbonates [15; 64].

**Summary Points**

Surficial authigenic carbonates are widespread in the deep sea, occurring both at active methane seeps and over extensive areas of past seepage. Often overlooked, these substrates host a distinctive fauna with a broad range of feeding modes. At methane seeps the level of activity, habitat matrix (hard vs soft substrate) and biogenic structures exert a major influence on faunal composition of carbonates. Gastropods and polychaetes are dominant at active sites, with crustaceans and echinoderms more common at inactive sites. Microbial grazing, of both bacteria and archaea is prevalent, with lithivory (grazing on the rock itself) occurring among neolepetopsid limpets and chitons. Both inactive and active carbonates host invertebrates with a range of chemosynthesis-based nutritional sources. On the Costa Rica margin, macrofaunal faunal densities are lower, but diversity is higher on carbonates than found in adjacent seep sediments. Overall, the substrate and nutritional heterogeneity introduced by seep carbonates contribute substantially to the diversity of macrofauna on continental margins. The extent to which the macrofaunal assemblages on the authigenic carbonates described here resemble those on the many other forms of carbonate crusts, platforms, scarps and rocks in the deep sea remains to be determined.

## Supporting Information

S1 Table Macrofaunal densities on authigenic carbonates by site and seepage activity. Mean per 200 cm^2^, Standard Error and percent of the total.
(XLSX)

S2 Table Macrofaunal densities on authigenic carbonates as a function of habitat for rocks sampled at depths > 900 m (at Mound 11, 12, Mound Quepos and Jaco Scar). Mean per 200 cm^2^, Standard Error and Percent of the Total.
(XLSX)

S3 Table Macrofaunal densities in active sediments as a function of habitat for tube cores sampled (at Mound 12, 11, Quepos Landslide and Jaco Wall). Mean per 200 cm^2^, Standard Error and Percent of the Total.
(XLSX)
