# Correction: Biodiversity on the Rocks: Macrofauna Inhabiting Authigenic Carbonate at Costa Rica Methane Seeps

**DOI:** 10.1371/journal.pone.0136129

**Published:** 2015-08-14

**Authors:** Lisa A. Levin, Guillermo F. Mendoza, Benjamin M. Grupe, Jennifer P. Gonzalez, Brittany Jellison, Greg Rouse, Andrew R. Thurber, Anders Waren

Fig 2, “Temperature and dissolved oxygen profile generated near Jaco Wall, Costa Rica from a CTD cast,” is incorrect and is a duplication of Fig.1, “Carbonates formations in different habitats on the Costa Rica Margin.” The authors have provided a corrected version here.

**Fig 2:**
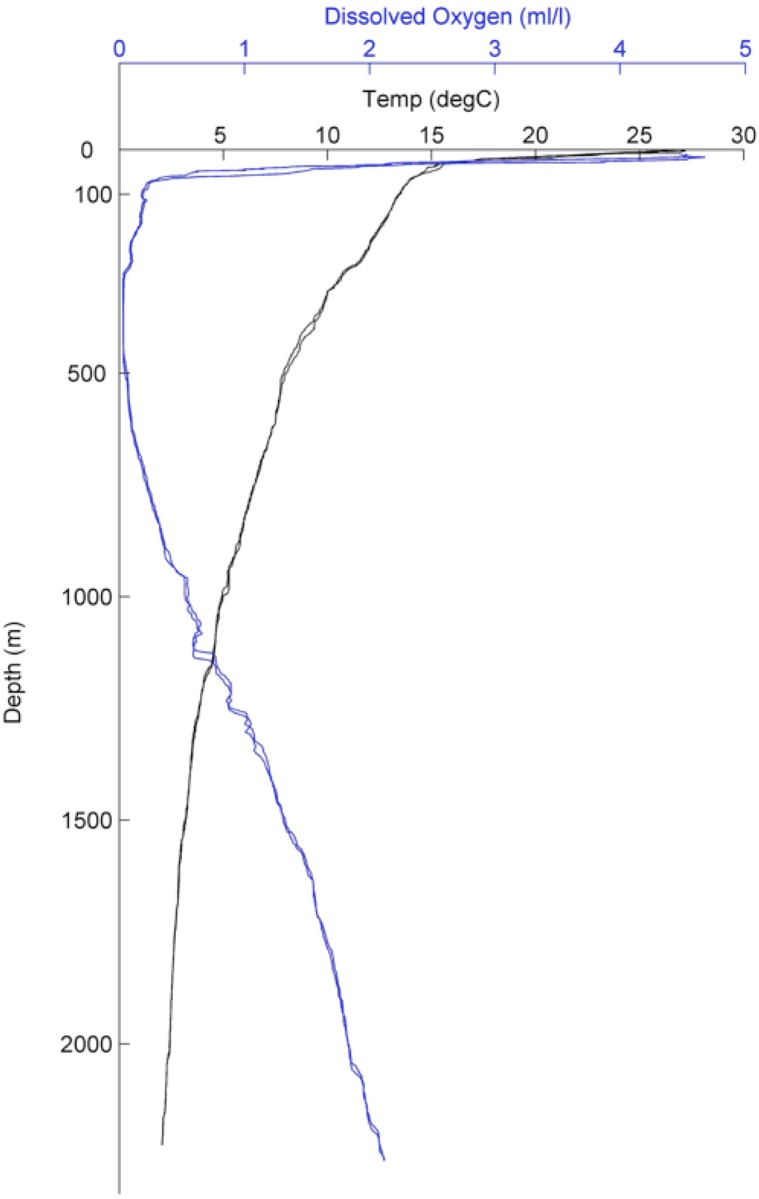
Temperature and dissolved oxygen profile generated near Jaco Wall, Costa Rica from a CTD cast.

Fig 11 is incorrect. Panels A and B were inadvertently switched. Panel B should be Panel A, and Panel A should be Panel B. The authors have provided a corrected version here.

**Fig 11:**
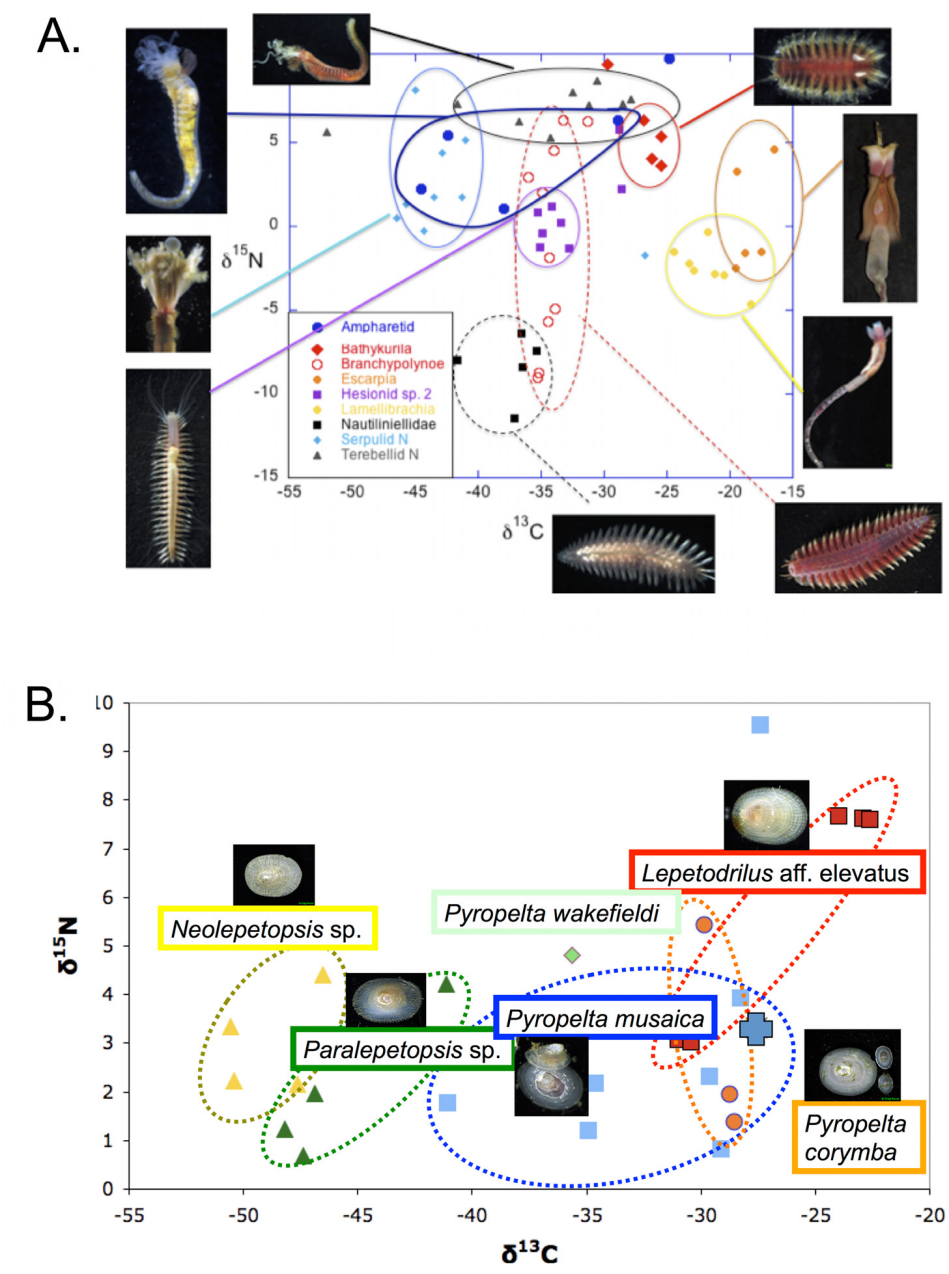
A. Dual isotope plot for polychaetes collected from carbonates on Mound 11 and 12 during AT 15–44. Costa Rica; B. Dual isotope plot for six limpet species collected on a single rock on Mound 12. In both figures each point represents a single individual.

There are errors in Table 6. The mean range columns have errors in the column titles. The authors have provided a corrected version here.

**Table 6:** Community isotope metrics for carbonate faunas on the Costa Rica Margin.

			Mean Distance to Centroid		Mean Nearest Neighbor		Mean Range δ^13^C		Mean Range δ^15^N		SEA		SEAc		Total Hull Area	
Activity		Number	mean	SE	mean	SE	mean	SE	mean	SE	mean	SE	mean	SE	mean	SE
	**Active**	**16**	5.5	0.9	3.4	0.8	-30.5	1.8	4.6	1.2	61.1	15.8	71.1	19.2	148.7	51.9
	**Inactive**	**5**	6.0	1.5	3.0	0.4	-25.1	3.4	9.1	1.9	51.4	30.3	15.7	9.6	36.2	21.9
**Site**																
	**Jaco Scarp**	**3**	1.7	0.8	1.3	0.6	-19.0	0.5	11.3	1.4	9.4	6.4	11.9	7.9	10.7	7.6
	**Mound 11**	**3**	6.4	1.9	2.5	0.3	-29.3	4.8	5.6	1.9	70.9	31.8	77.3	33.9	132.2	52.7
	**Mound 12**	**10**	6.3	1.1	4.1	1.0	-34.0	1.7	2.8	1.4	69.4	19.0	72.8	24.8	179.4	81.1
	**Mound Quepos**	**4**	5.3	2.1	3.5	1.6	-25.1	3.2	8.6	2.6	62.7	52.2	55.3	46.6	98.7	84.3
	**Quepos Landslide**	**1**	9.9		3.1		-28.8		6.4							
**Active Habitat**																
	**Bacterial Mat**	**3**	6.4	2.0	3.9	1.7	-32.6	2.1	6.2	1.7	55.8	39.3	63.7	40.3	96.8	71.4
	**Mussel Bed**	**8**	5.3	1.3	3.3	1.3	-32.9	2.5	3.6	1.9	59.1	20.7	71.1	29.1	139.8	67.7
	**Tubeworms**	**3**	7.3	3.0	4.4	1.9	-29.7	3.8	2.1	2.4	102.7	50.9	114.2	56.8	310.1	191.9
	**Jaco Rocks**	**2**	2.4	0.5	1.8	0.5	-19.3	0.7	10.2	1.5	14.0	7.6	17.7	9.2	16.1	9.3

There are errors in Table 7. A few of the coordinates in the Latitude/Longitude column are not formatted correctly. Please see a corrected version here.

**Table 7:** Macrofaunal densities on hard substrates in the deep sea and shallow waters.

Substrate	Location	Water Depth (m)	Latitude/Longitude	Density/unit area	Density ind./200cm^2^	# individuals	# species	Surface area	Dominant taxa	Reference
Manganese nodules	equatorial and central North Pacific	4500–5800	5°N, 125°W 30°N, 157°W	1090 ind./m^2^	21.8	120	32	0.11 m^2^		Mullineaux (1987)
Whale skeleton	San Nicolas	960	33°20'N, 119°59'W	6169 ind./m^2^	123.38	5120	190	0.83 m^2^	Bivalvia	Baco and Smith (2003)
Whale skeleton	San Catalina Basin	1240	33°12'N, 118°29'W	16375 ind./m^2^	327.5	20632	180	1.26 m^2^	Bivalvia	Baco and Smith (2003)
Whale skeleton	San Clemente Basin	1910	32°26'N, 118°9'W	11005 ind./m^2^	220.1	11555	102	1.05 m^2^	Bivalvia	Baco and Smith (2003)
Vent Mussel Beds	Mid-Atlantic Ridge	1600	37°17'N, 32°16'W	811 ind./L of mussel		20044	25	24.7 L of mussel	Crustacea	Van Dover and Trask (1999)
Deep-sea rocks	San Nicolas	960	33°15'N, 119°56'W	490 ind./m^2^	9.8	147	26	0.3 m^2^		Baco and Smith (2003)
Seamount	Davidson	1246–3289	35°43'N 122°43'W	0.87 ind./m^2^	0.0174	59933	148		Cnidaria	Lundsten *et al*. (2009)
Seamount	Pioneer	811–1815	37°21'N, 123°26'W	2.19 ind./m^2^	0.0438	36430	110		Cnidaria	Lundsten *et al*. (2009)
Seamount	Rodriguez	619–2120	34°01'N, 121°04'W			38087	133		Echinodermata	Lundsten *et al*. (2009)
Sponge stalks	Station M	4100	34°45'N, 123°00'W	17572 ind./m^2^	351.44	1933	104	0.11 m^2^	Polychaeta	Beaulieu (2001)
Wood	Haakon Mosby Mud volcano	1257	72°00'N, 14°43'E	14988 ind./dm^3^	299.76	2398			Bivalvia	Gaudron *et al*. (2010)
Rocky Shore	Australia-Tropical (exposed/sheltered)	intertidal	23°S 151°E	97.6/ 31.5 per 400 cm^2^	49/16		12/14.8	400 cm^2^	Cirripedia	73
Rocky Shore	New Zealand—Temperate (exposed/sheltered)	Intertidal	45°S 170°E	265/64.8 per 400 cm^2^	133/33		12/15.2	400 cm^2^	Cirripedia	73
Mussel beds	Eagle Island Alaska	0	54°62′N, 159°99′W	970 ind./L of mussel		78353	70	80.7 L of mussel	Polychaeta	Van Dover and Trask (1999)
Rocky shore	South-Central California (early/mid/late succession)	intertidal algal mats on boulders	34°25'N 119°41'W	78/316/294 per 0.01m^2^	156/632/588		214	0.09 m^2^	Crustacea/ Polychaeta	75
